# Open-Vessel and Scalable Synthesis of Linear and Branched Poly(meth)acrylic Acid via Light-Mediated Atom Transfer Radical Polymerization in Water

**DOI:** 10.1021/acs.macromol.5c00952

**Published:** 2025-06-11

**Authors:** Arman Moini Jazani, Kriti Kapil, Hironobu Murata, Mozhdeh Madadi, Julian Sobieski, Piotr Mocny, Khidong Kim, Roberto R. Gil, Krzysztof Matyjaszewski

**Affiliations:** Department of Chemistry, 6612 Carnegie Mellon University, 4400 Fifth Avenue, Pittsburgh, Pennsylvania 15213, United States

## Abstract

Poly­(methacrylic acid) (PMAA) and poly­(acrylic acid) (PAA) are synthesized on a large scale by conventional free radical polymerization (FRP). The access to architectural diversity by FRP is limited but can be addressed by reversible deactivation radical polymerization (RDRP), including atom transfer radical polymerization (ATRP). ATRP of methacrylic acid (MAA) and acrylic acid (AA) is challenging due to lactonization via the displacement of halide (*X*) end groups by penultimate carboxylate anions (CO_2_^–^) and the loss of chain-end functionality. Despite the successful polymerization of MAA or AA ((M)­AA) using various RDRP methods, the oxygen-tolerant photo-ATRP of (M)­AA has not yet been investigated. Herein, photo-ATRP of (M)­AA in open vials was enabled by adding sodium pyruvate (SP) or pyruvic acid (PA) to the polymerization mixture. Photoirradiation of SP/PA generated radicals and enhanced the rate of polymerization at ambient temperature, which diminished lactonization reactions. This method allowed the synthesis of PMAA or PAA (P­(M)­AA) at low pH (1 to 3.2) with relatively low dispersity (*Đ* = 1.10–1.38 under optimized conditions) and good agreement between the theoretical molecular weight (*M*_n,theo_) and the absolute molecular weight (*M*_n,abs_). Photo-ATRP allowed the synthesis of PMAA in ≤1 h and also the synthesis of branched PAA by copolymerization with α-haloacrylic acids. Additionally, successful grafting of MAA from poly­(vinylidene fluoride-*co*-chlorotrifluoroethylene) (PVDF-*co*-CTFE) was achieved in dispersed aqueous media.

## Graphical abstract

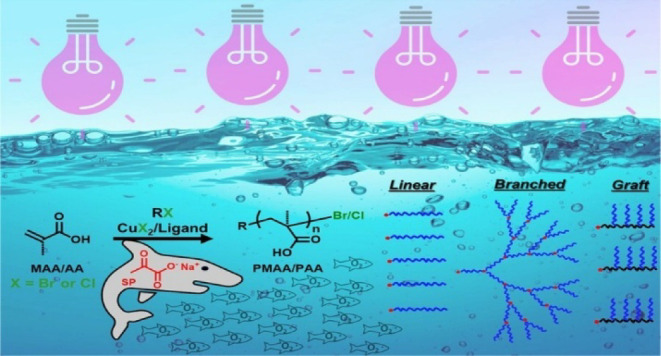

## Introduction

Poly­(methacrylic acid) (PMAA) and poly­(acrylic acid) (PAA) are important polymers with various applications, such as superabsorbent polymers, dispersants, thickeners, flocculants, wound-healing dressings, antifouling surfaces, stimuli-responsive materials, and drug delivery vehicles.^1–7^ PMAA and PAA are typically synthesized by homogeneous or heterogeneous free radical polymerization (FRP) of methacrylic acid (MAA) or acrylic acid (AA) in water.^8–10^ The limited control of polymer architecture and the broad molecular weight distribution of polymers synthesized by FRP have fostered the quest for alternative strategies. Reversible deactivation radical polymerization (RDRP), also known as controlled radical polymerization (CRP), has provided a considerable paradigm shift in modern synthetic polymer chemistry for the synthesis of well-controlled polymers.^11–13^ The two most common RDRP methods include atom transfer radical polymerization (ATRP) and reversible addition–fragmentation chain- transfer polymerization (RAFT).^14,15^ RDRP enables macromolecular engineering via precise control of molecular weight, dispersity, chain-end functionality, and polymer architecture.^16,17^ ATRP employs transition metal complexes (*e.g.,* Cu, Fe, and Ru) to intermittently activate alkyl halides (*R*–*X*) and generate carbon-centered radicals.^18,19^ The key to ATRP synthesis of well-defined polymers is to maintain a dynamic equilibrium that favors radical deactivation to diminish irreversible radical termination and provide concurrent growth of all polymer chains.^20–22^

While PMAA and PAA (P­(M)­AA) were efficiently synthesized by RAFT polymerization,^23–26^ the controlled polymerization of MAA and AA ((M)­AA) by ATRP has been more challenging, mainly due to the lactonization reaction and the displacement of the halide end group by penultimate carboxylate anions (COO^–^) (Figure 1A).^27^ Catalyst poisoning via the displacement of metal halide ligands by (M)­AA, as well as the protonation of amine-based ligands, further complicated the polymerization of (M)­AA by ATRP (Figure 1A).^28–30^ Therefore, the synthesis of P­(M)­AA by ATRP was often carried out by using protecting groups (e.g., *tert*-butyloxycarbonyl protecting group) in organic solvents (i.e., anisole, 1,4-dioxane, dimethylformamide, toluene, or dimethyl sulfoxide),^31–34^ followed by subsequent deprotection using strong acids (i.e., trifluoroacetic acid or hydrochloric acid). The polymerization of sodium methacrylate (the sodium salt of methacrylic acid) or amine salts of methacrylic acids was also reported, albeit with limited control.^35–37^ Some efforts were devoted to carrying out the direct ATRP of (M)­AA in its acidic (nonionic) form, employing normal ATRP,^38^ supplemental activation reducing agent (SARA),^39^ electrochemical,^27^ iron-catalyzed,^40^ surface-initiated (SI),^41^ and organocatalyzed ATRP.^42^ Most techniques required a highly acidic environment, had low initiation efficiency (I*), and formed polymers with relatively high dispersity. Nevertheless, some lessons from these studies were gained, which paved the way for future optimization of ATRP conditions. Importantly, enhancing control in polymerization and surmounting lactonization could be achieved by using chlorine (Cl) rather than bromine (Br) as the end group, accelerating the rate of polymerization, and increasing the acidity (pH < 2), which decreased the nucleophilicity of the carboxylic moiety.^27^

**Figure 1:**
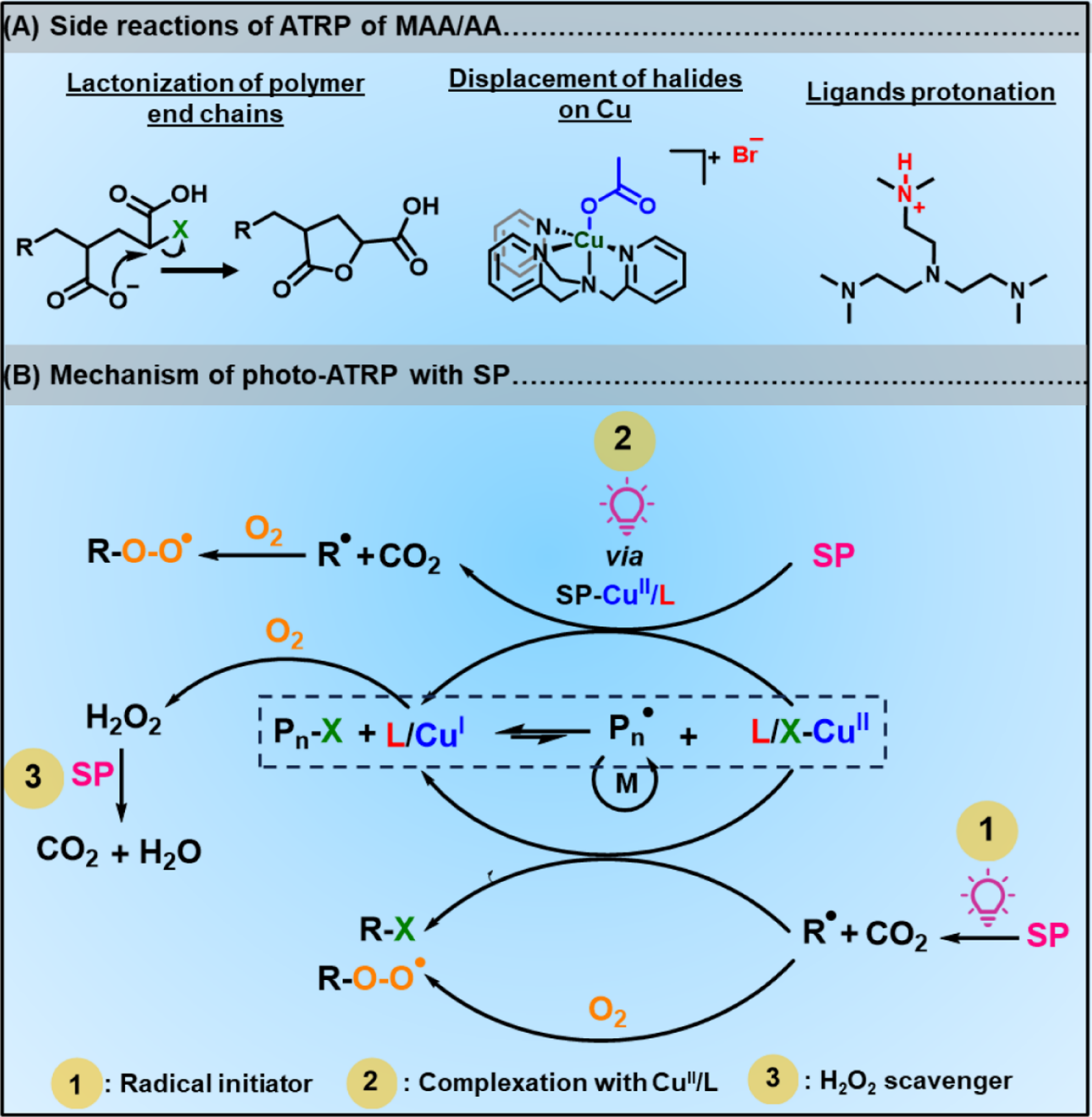
(A) Possible side reactions in ATRP of (M)­AA. (B) Mechanism of oxygen-tolerant photo-ATRP with sodium pyruvate (SP).

Recently, the use of light to mediate ATRP has become a growing field of research, leading to the development of methods such as copper and iron photo-ATRP,^43–45^ organocatalyzed ATRP,^46–48^ and dual metal/photoredox catalyst (PC) ATRP.^49–57^ Photomediated ATRP has all the merits of conventional ATRP (i.e., wide monomer scope, low catalyst loading, tolerance to impurities), plus the benefits of polymerization at ambient temperature, improved energy efficiency, and spatial and temporal control.^58–60^ Various efforts were made or are underway to expand the monomer scope of photo-ATRP.^61,62^ However, photo-ATRP for the synthesis of PMAA and PAA homopolymers has not yet been investigated. Some attempts to copolymerize MAA and AA with other hydrophobic monomers (i.e., benzyl methacrylate and styrene) using iridium PCs or benzophenone photoradical initiators in organic solvents (i.e., DMF) were reported.^63,64^ In addition, photo-ATRP can have exceptional oxygen tolerance due to the rapid photoinduced generation/regeneration of catalysts (i.e., PCs or metal complexes), which catalyze the conversion of triplet O_2_ to other reactive oxygen species (superoxide anion, H_2_O_2_, and hydroxyl radicals).^65,66^ The synthesis of P­(M)­AA without conventional degassing procedures (i.e., N_2_ sparging or freeze–pump–thaw procedures) could facilitate experimental setup and enhance the expansion of method to large-scale polymerization in industry.

Our group has recently reported that the addition of sodium pyruvate (SP), a common intermediate of human metabolism and a cell culture component, to photo-ATRP offers significant merits, including enhanced oxygen tolerance, acceleration of the polymerization rate, and the ability to polymerize challenging monomers (*e.g.,* acrylamide) at ambient temperature.^67,68^ These advantages stem from the multiple roles of SP in ATRP (Figure 1B): 1) SP photodecomposes in the presence of light and acts as a photoradical initiator, generating radicals (R^•^);^69^ thereby, ATRP driven by SP was termed photoinitiators for continuous activator regeneration (PICAR-ATRP).^67^ 2) SP coordinates with Cu^II^/L complexes and promotes the photoreduction of the pyruvate-Cu^II^/L to Cu^I^/L complex by ligand-to-metal charge transfer (LMCT), followed by decarboxylation. 3) SP prevents new chain formation from hydroxyl radicals (OH**•**) (generated via the Fenton reaction) by scavenging H_2_O_2_ produced in oxygen-tolerant photo-ATRP, generating CO_2_, H_2_O, and sodium acetate (SA).^70,71^ The combination of radical flux generated by SP and Cu^I^/L formation provides ATRP with excellent oxygen tolerance.^72^

In the pursuit of more sustainable polymerizations of (M)­AA that adhere to green chemistry principles, we applied PICAR-ATRP to synthesize P­(M)­AA in water. We hypothesized that the faster polymerization of (M)­AA, due to the addition of SP and the higher propagation rate constants of (M)­AA in their acidic form (several times higher than the ionized form),^73^ as well as the ambient temperature conditions of photo-ATRP, can strongly reduce the rate of the intramolecular lactonization reaction, thus minimizing the loss of chain-end Cl/Br. In addition, PICAR-ATRP confers the benefit of polymerizing (M)­AA in a completely open-cap vial without prior deoxygenation for the first time. We also applied our method to synthesize novel architectures of P­(M)­AA, such as branched or graft polymers from poly­(vinylidene fluoride-*co*-chlorotrifluoroethylene) (PVDF-*co*-CTFE), without derivatization (protection/deprotection) in aqueous dispersed media.

## Results and Discussion

### Synthesis of Linear PMAA by PICAR-ATRP with HOBiB as the Initiator

Photo-ATRP of MAA was performed in an open-cap vial (without stirring) under irradiation with LED UV light (380 nm, 28.5 mW/cm²) in a photoreactor equipped with an in-built fan that maintained a low temperature (<24 °C) during the polymerization. The first experiments were carried out using CuBr_2_/tris­(2-pyridylmethyl)­amine (TPMA) as the catalyst and 2-hydroxyethyl α-bromoisobutyrate (HOBiB) as the initiator (Figure 2). The molar ratio of components used for polymerization was MAA/initiator/Cu*X*_2_/TPMA/SP = 200/1/0.2/0.6/65. To inhibit the dissociation of the complex [*X*–Cu^II^/L (Ligand)]^+^ into a free halide anion *X* ^–^ and [Cu^II^/L]^2+^, which is an inefficient deactivator, PBS (prepared with NaCl, 10% *v/v*) was added to the polymerization mixture.^74^ DMSO (10% *v/v*) was also used to increase the solubility of the Cu^II^/L complex and to scavenge reactive singlet oxygen species produced during aerated ATRP.^65^ The addition of MAA rendered the polymerization mixture more acidic (pH = 3.2), and the addition of SP (p*K*_a_ = 2.9) did not result in the transformation of MAA (p*K*_a_ = 4.6) into sodium methacrylate (Figure S1). All polymerizations were carried out using purified TPMA to prevent interference from other photoactive impurities.^75^

**Figure 2:**
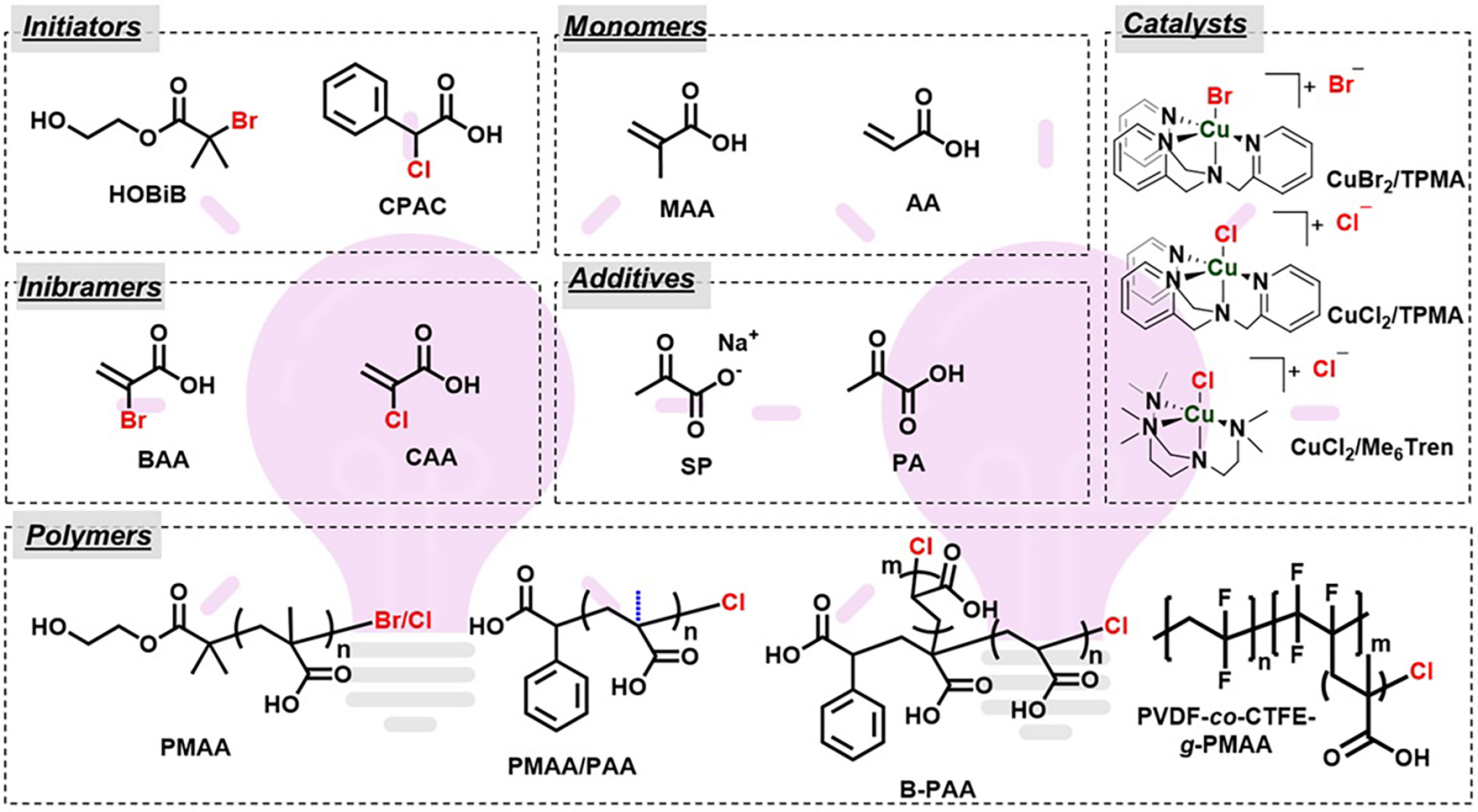
Chemical structures of initiators, monomers, inibramers, additives, and catalysts used for photo-ATRP of (M)­AA and the resulting polymers from the polymerizations. HOBiB: 2-hydroxyethyl α-bromoisobutyrate; CPAC: 2-chloro-2-phenylacetic acid; MAA: methacrylic acid; AA: acrylic acid; BAA: 2-bromoacrylic acid; CAA: 2-chloroacrylic acid; SP: sodium pyruvate; PA: pyruvic acid; TPMA: tris­(2-pyridylmethyl)­amine; Me_6_TREN: tris­[2-(dimethylamino)­ethyl]­amine.

Under classical photo-ATRP conditions (without SP, black squares, Figure 3A) or in the absence of light (entries 1 and 2, Table 1), no conversion of MAA was observed within 45 min. However, the introduction of SP promoted rapid polymerization, reaching 85% conversion of MAA (entry 3, Table 1; red squares, Figure 3A). The absolute molecular weight (*M*_n,abs_) of the PMAA was in good agreement with the theoretical molecular weight (*M*_n,theo_ *)* and the size exclusion chromatography (SEC) trace was monomodal (Figure S2), although the dispersity was high (*Đ* = 1.60). In an attempt to further increase the rate of polymerization and minimize lactonization, the SP concentration was doubled. This change did not yield faster polymerization (green squares, Figure 3A), albeit it was accompanied by decreased dispersity of PMAA (*Đ* = 1.47; entry 4, Table 1), which could be due to a small change in the pH of the polymerization solution. This suggests that the photoradical production of SP reaches a plateau after a certain photoirradiation time and does not increase with higher concentrations of SP, as reported in the literature.^76^

**Table 1:** Synthesis of Linear PMAA Homopolymers by PICAR-ATRP of MAA with HOBiB Initiator^a^

Entry	Notes	Cu*X*_2_	pH	^b^Conv. (%)	^c^ *M*_n,theo__(×1000)_	^d^ *M*_n,abs__(×1000)_	^d^ *Đ*	^e^I* (%)
1	No SP	CuBr_2_	3.2	<5	-	-	-	-
2	No light	CuBr_2_	3.2	<5	-	-	-	-
3	65 eqv SP	CuBr_2_	3.2	85	18.3	17.0	1.60	108
4	135 eqv SP	CuBr_2_	3.2	82	17.7	18.3	1.47	97
5	65 eqv SP	CuCl_2_	3.2	83	17.9	22.6	1.38	79
6	65 eqv SP	CuCl_2_	1.2	85	18.4	36.0	1.16	51

a Reactions conditions: [MAA]_0_/[HOBiB]_0_/[Cu*X*_2_]_0_/[TPMA]_0_/[SP]_0_ = 200/1/0.2/0.6/65 in H_2_O with DMSO (10% *v*/*v*) and PBS (10% *v/v*), irradiated with Evoluchem LEDs (380 nm, 28.5 mW/cm^2^) in an open-cap vial at 1 mL scale (without stirring) for 45 min. [MAA]_0_ = 1742 mM.

b Monomer conversion was determined by using ^1^H NMR spectroscopy.

c *M*_n,theo_ were calculated using molecular weight of sodium methacrylate.

d *M*_n,abs_ and dispersity (*Đ*) were determined by size exclusion chromatography analysis with a multiangle light scattering detector (SEC-MALS) using aqueous NaCl/PBS (pH = 8) as the eluent.

e *M*_n,theo_/*M*_n,abs_.

**Figure 3:**
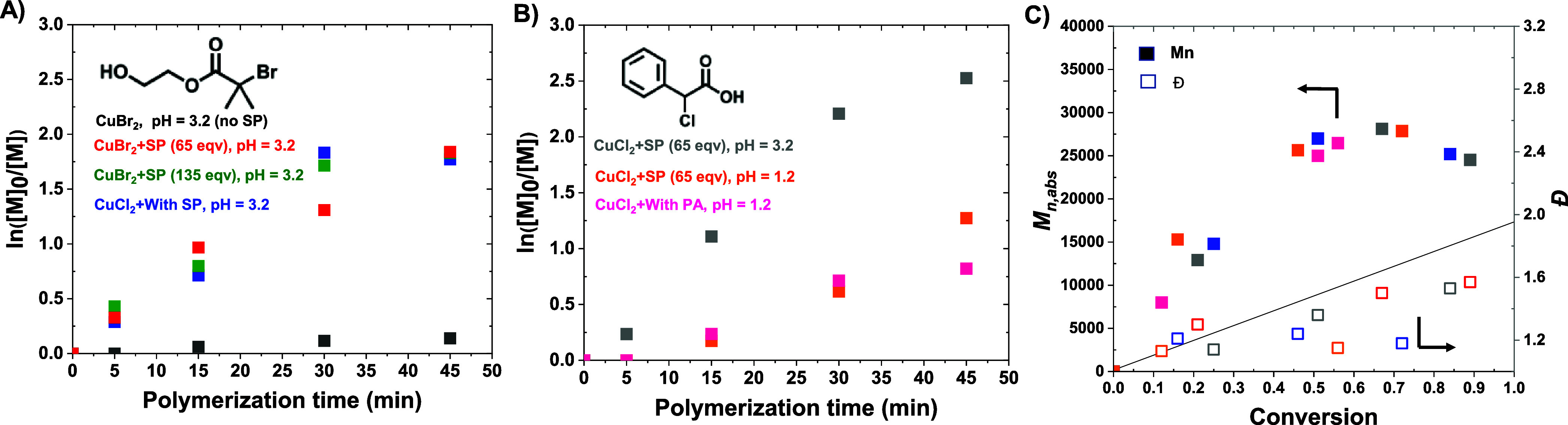
Kinetics of PICAR-ATRP of MAA with (A) HOBiB and (B) CPAC initiators, and (C) evolution of absolute molecular weight (*M*_n,abs_) and dispersity (*Đ*) over conversion.

Replacing CuBr_2_ with CuCl_2_ did not have a significant effect on the polymerization rate (blue squares, Figure 3A) and dispersity of PMAA (*Đ* = 1.38; entry 5, Table 1). This is because most chains still contain Br end groups when polymerization is initiated from HOBiB (≈83% Br and 17% Cl end-chain with the molar ratio of HOBiB/CuCl_2_ = 1/0.2 in the feed). The initiation efficiency (I*, *M*_n,theo_/*M*_n,abs_) was slightly compromised when CuCl_2_ was used (79% vs 108% for CuCl_2_ and CuBr_2_). This could be due to the slower *R*–*X* activation of Cl vs Br chain ends. Reducing the pH of the polymerization solution by the addition of HCl (pH = 1.2) significantly decreased the dispersity of PMAA (*Đ =* 1.16), suggesting the improved preservation of the chain ends due to the suppressed lactonization (entry 6, Table 1). The peaks of lactone were not detectable in ^1^H NMR, due to the high molecular weight of PMAA synthesized by PICAR-ATRP. However, the formation of lactone was comprehensively demonstrated in previous work.^27^

The polymerization in the presence of poly­(ethylene glycol) (PEG) ATRP macroinitiator (PEG-bromide, *M*_n_ = 2166 g/mol) resulted in the precipitation of polymers after ≥10 min of irradiation (Figure S3). The precipitated polymer could be redissolved in water after the addition of sodium bicarbonate, suggesting that the precipitation was not due to cross-linking.^8^ This indicated that the hydrophobicity of polymers from the PEG macroinitiator was higher than that of CPAC and HOBiB initiators, since PEG has both hydrophilic and hydrophobic properties.^77^

### Synthesis of Linear P­(M)­AA by PICAR-ATRP with the CPAC Initiator

The polymerization using a more active Cl-based ATRP initiator, 2-chlorophenylacetic acid (CPAC), did not improve the I* and dispersity compared to the less active HOBiB initiator (entry 1, Table 2). However, the polymerization resulted in a moderately faster rate (gray squares, Figure 3B) and reached higher conversions (89%). Similar to HOBiB, reducing the pH of the polymerization solution (pH = 1.2) led to a narrower molecular weight distribution of PMAA (*Đ* = 1.17, entry 2, Table 2). The I* decreased upon reducing the pH (79% vs 56% at pH = 3.2 and pH = 1.2, respectively), which could be due to the reduced activity of the CuCl_2_ catalyst under acidic conditions (+0.12 V lower for CuBr_2_/TPMA when changed from pH = 7 to 2.2).^40^ The difference in the activity of the catalyst at different pH levels was also evident in the kinetics of polymerization (gray and orange squares in Figure 3B), in which the polymerization was considerably slower and had an induction period (5 min) under acidic conditions. The polymerization at more diluted conditions (3-fold lower concentrations of all components, entry 3, Table 2) was successful and reached high conversion (93%) but at a longer irradiation time (165 min). Polymerization afforded PMAA with a broader molecular weight distribution (*Đ =* 1.48) and lower values of I* (45%). The ^1^H NMR analysis of residual SP upon light irradiation indicated that ≥88% of SP remains intact in ATRP (Figure S4). The polymerization of MAA at a lower SP concentration (8 or 16 mM) was also possible and reached high conversion, although it required a longer polymerization time (Table S1).

**Table 2:** Synthesis of PMAA and PAA Homopolymers by PICAR-ATRP of MAA (Entries 1–4) and AA (Entries 5–6) with the CPAC Initiator^a^

Entry	Monomer	Ligands	pH	Time (min)	^b^Conv. (%)	^c^ *M*_n,theo__(×1000)_	^d^ *M*_n,abs__(×1000)_	^d^ *Đ*	^e^I* (%)
1	MAA	TPMA	3.2	30	89	19.4	24.5	1.57	79
2	MAA	TPMA	1.2	45	72	15.7	27.8	1.17	56
3^f^	MAA	TPMA	1.2	165	93	16.2	36.2	1.48	45
4^g^	MAA	TPMA	1.4	30	51	11.2	25.0	1.10	45
5	AA	Me_6_TREN	3.2	60	57	8.3	12.6	1.28	66
6	AA	Me_6_TREN	1.2	60	39	5.8	12.0	1.42	48

a Reactions conditions: [MAA or AA]_0_/[CPAC]_0_/[CuCl_2_]_0_/[Ligand]_0_/[SP]_0_ = 200/1/0.2/0.6/65 in H_2_O with DMSO (10% *v*/*v*) and PBS (10% *v/v*), irradiated with Evoluchem LEDs (380 nm, 28.5 mW/cm^2^) in an open-cap vial at 1 mL scale (without stirring). [MAA]_0_ = 1742 mM.

b Monomer conversion was determined by using ^1^H NMR spectroscopy.

c *M*_n,theo_ were calculated using molecular weight of sodium methacrylate/sodium acrylate.

d *M*_n,abs_ and *Đ* were determined by size exclusion chromatography analysis with a multiangle light scattering detector (SEC-MALS) using aqueous NaCl/PBS (pH = 8) as the eluent.

e *M*_n,theo_/*M*_n,abs_.

f Under diluted conditions: [MAA]_0_ = 580 mM, [CPAC]_0_ = 2.9 mM, [CuCl_2_]_0_ = 0.58 mM, [TPMA]_0_ = 1.7 mM, and [SP]_0_ = 100 mM.

g PA was used instead of SP.

The replacement of SP with pyruvic acid (PA, the nonionic form of SP, p*K*_a_ = 2.4) resulted in similar kinetics to the polymerization with SP at acidic pH (entry 4, Table 1; pink squares, Figure 3B). This is because the addition of PA made the polymerization solution more acidic (pH = 1.4) and eliminated the need for further adjustment by HCl. Furthermore, the polymers remained equally oxygen-tolerant, suggesting the possibility of further expanding of this approach to other α-keto esters/carboxylates (e.g., phenyl glyoxylic acid, ethyl pyruvate, or oxalic acid).^78^ Interestingly, polymerization with PA outperformed SP with regard to molecular weight distribution (*Đ* = 1.10), which was confirmed by repeating the experiment and obtaining identical results. This can be explained by the fact that sodium carboxylates (i.e., SP) may replace halide anions in the CuCl_2_/L complex and convert them into less efficient Cl–Cu^II^/L-SP deactivators.

PICAR-ATRP of AA was also investigated using CPAC and CuCl_2_, with tris­[2-(dimethylamino)­ethyl]­amine (Me_6_TREN) as a ligand to form a more active ATRP catalyst. The molar ratios of AA/CPAC/CuCl_2_/Me_6_TREN/SP = 200/1/0.2/0.6/65 were used. ATRP of AA is more challenging than MAA due to the faster lactonization of secondary alkyl halide end groups. However, PAA with a narrow molecular weight distribution (*Đ* = 1.28) and acceptable conversion (57%) was obtained after 60 min of irradiation at pH = 3.2 (entry 5, Table 2). To diminish the lactonization of PAA-Cl, ATRP of AA was carried out under more acidic conditions (pH = 1.2). More acidic conditions resulted in lower I* and a broader molecular weight distribution (*Đ* = 1.42, entry 6, Table 2), unlike MAA, where the acidic conditions reduced dispersity. This can be related to the protonation of alkyl amines in Me_6_TREN, which are more basic than pyridines in TPMA and may result in the formation of ligand-free (unbound) CuCl_2_.

The control in the polymerization of MAA using PICAR-ATRP was confirmed by a linear first-order kinetic plot and a linear increase in polymer molecular weight with conversion (Figure 3A–C). However, after reaching a conversion of >60%, deviation from the linear semilogarithmic kinetic plot was observed. This could suggest increased lactonization or radical termination reactions, which reduced the constant concentration of radicals at higher conversions. In addition, the formation of new radical-initiating species from carboxylic acids in photomediated conditions in the presence of Cu could yield low molecular weight polymer chains and reduce the final *M*_n,abs_ (*vide infra*). Despite the deviation from linear first-order kinetics at higher conversion, SEC traces for all final polymers were monomodal (Figure S2).

### Investigation of Side Reactions in Photoinduced ATRP of MAA

The use of UV light in ATRP of MAA could induce some side reactions. Three side reactions are particularly important: 1) Supplemental activation of *R*–*X* via interaction of CO_2_^–^ of (M)­AA and *R–X*, followed by subsequent photoreductive cleavage of the *C*–*X* bond. 2) Complexation of CO_2_^–^ with Cu^II^/L, followed by decarboxylative LMCT in a manner similar to SP. 3) Replacement of *R*–*X* chain ends by SP due to the higher concentrations of SP compared to *R*–*X*.

The reaction of MAA with *R*–*X* (without Cu^II^/L) was first investigated under irradiation with UV light.^79–81^ UV irradiation of the mixture of MAA and two alkyl halide initiators (i.e., HOBiB and CPAC) led to the formation of highly viscous polymer solutions, which were poorly soluble in typical NMR solvents (i.e., DMSO-*d*_6_ or D_2_O), whereas no polymerization occurred without UV irradiation. This unexpected synergistic interaction of alkyl halides and ionized MAA, along with the formation of polymers with very high molecular weights due to the generation of a low concentration of radicals via photocleavage of the C–Cl bond, could suggest some involvement of halogen bonding.^80,81^ The conversion of carboxylate anions (COO^–^) to carboxylic acids (COOH) by the addition of HCl (pH = 1) suppressed this reaction. Other control experiments confirmed the need of CPAC, MAA, and UV light for this reaction, excluding the possibility of direct homolytic cleavage of alkyl halides by light or photodegradation of MAA (Figure S5).^82–84^ The coordination of MAA with CuCl_2_/TPMA complex and subsequent photodecarboxylation of MAA under UV light was also investigated.^85–88^ The photodecarboxylation of MAA or PMAA with the Cu/L complex can result in the reduction of Cu^II^ to Cu^I^, formation of new radical species from MAA, or branching or degradation of PMAA by β-carbon fragmentation via the generation of midchain radicals.^89–91^ Therefore, the control in the polymerization of MAA can be affected by these events.^30,92^ Nevertheless, UV–vis spectroscopy revealed that the extent of this reaction was ≤8% (Figure S6). In addition, since both MAA and PMAA (p*K*_a_ = 4.8–5.3) are not in their ionic form during PICAR-ATRP (pH = 1–3.2), it is unlikely that their interactions with the Cu^II^/L complex would be significant enough to impede polymerization.

Finally, the presence of a large excess of SP could result in the formation of new radicals and the replacement of the *R*−*X* in PMAA. Filtered 1D ^1^H diffusion-ordered spectroscopy (DOSY) was employed to suppress the signals of fast-diffusing small molecules, enabling clearer observation of polymer-associated species.^93,94^ The analysis of purified PMAA demonstrated the presence of aromatic peaks from the CPAC initiator at 7.3 ppm (Figure 4), even after 40% filtered diffusion was applied, suggesting that aromatics are bound to the polymer chains and that CPAC acts as the initiator for the polymerization of PMAA.

**Figure 4:**
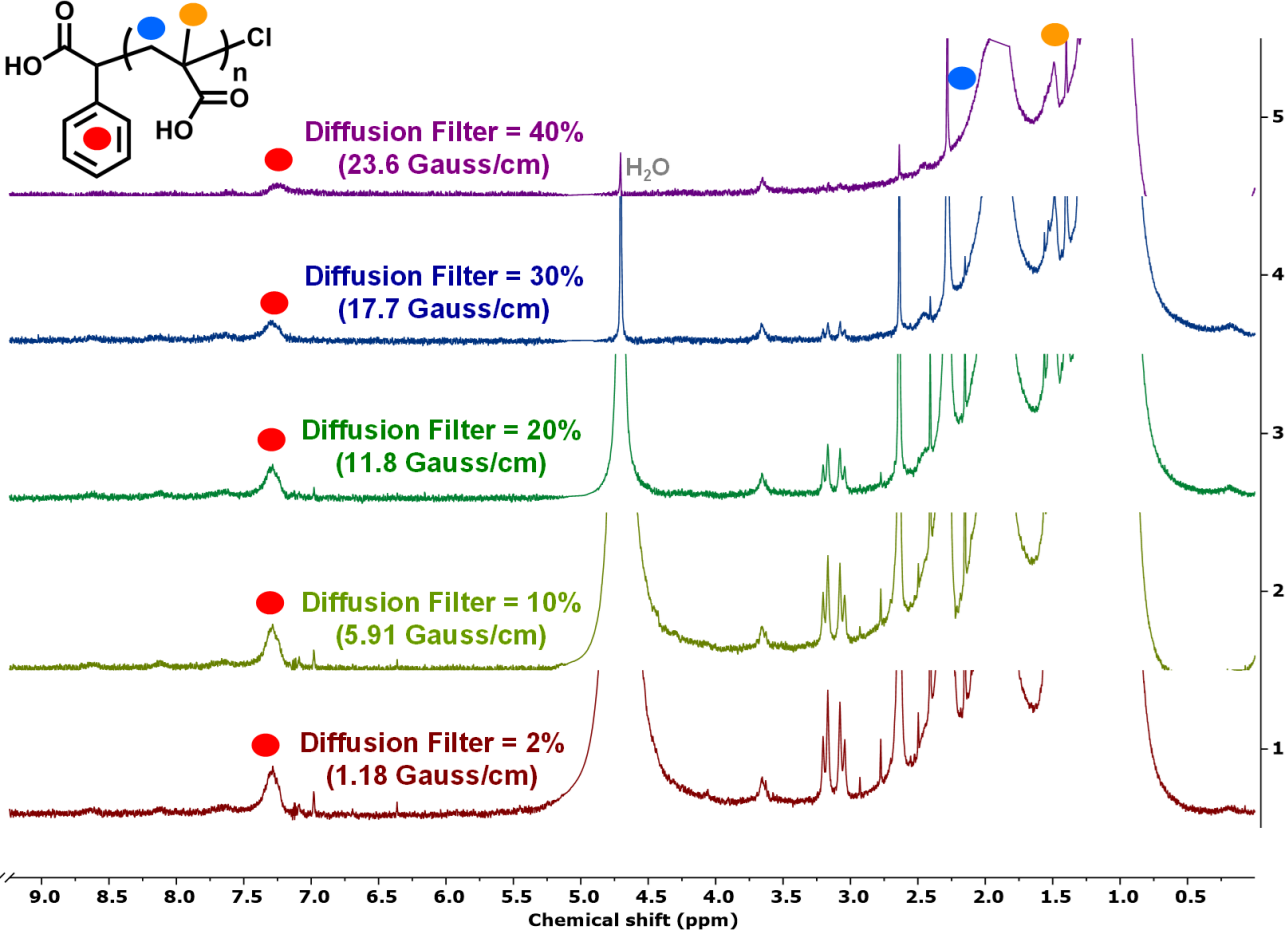
1D ^1^H diffusion-ordered spectroscopy of PMAA-Cl from the CPAC initiator in D_2_O, with a diffusion filter range of 2–40%.

### Scope of PICAR-ATRP of MAA

After the conditions were optimized, PICAR-ATRP was used to prepare PMAA with different molecular weights (Table 3, entries 1–5). PMAA with target degrees of polymerization (DP_target_ = 50, 100, 200, 400, and 800) was synthesized by changing CPAC concentrations while maintaining a constant concentration of all other components. High conversions (>50%) were reached for all DP_target_, and dispersity remained low (*Đ* < 1.35), except for DP_target_ = 50 (*Đ* = 1.57). SEC traces showed evolution to higher molecular weight with higher DP_target_ and remained monomodal in all cases (Figure 5A). However, for DP_target_ = 800, the SEC traces displayed a smaller shift to higher molecular weight, indicating that the limit of control was reached. I* improved as PMAA with higher DP_target_ was synthesized (I* = 19, 39, 56, 76, 113 for DP_target_ = 50, 100, 200, 400, and 800). This suggests termination events at high concentrations of alkyl halides (and radicals), slower deactivation, and, at lower concentrations of initiator, an increased contribution from the formation of new chains from radical sources other than *R*−*X*.

**Table 3:** Synthesis of Linear PMAA Homopolymers with Different DP_target_ by PICAR-ATRP of MAA^a^

Entry	DP_target_	^b^Conv. (%)	^c^ *M*_n,theo__(×1000)_	^d^ *M*_n,abs__(×1000)_	^d^ *Đ*	^e^I* (%)
1	50	75	4.06	20.9	1.57	19
2	100	80	8.64	22.2	1.30	39
3	200	72	15.7	27.8	1.17	56
4	400	70	30.3	39.7	1.25	76
5	800	54	46.7	41.4	1.26	113

a Reactions conditions: [MAA]_0_/[CPAC]_0_/[CuCl_2_]_0_/[TPMA]_0_/[SP]_0_ = 200/0.25–4/0.2/0.6/65 in H_2_O with DMSO (10% *v*/*v*) and PBS (10% *v/v*), irradiated with Evoluchem LEDs (380 nm, 28.5 mW/cm^2^) for 60 min (except entry 3, for 45 min) in an open-cap vial at 1 mL scale (without stirring). [MAA]_0_ = 1742 mM.

b Monomer conversion was determined by using ^1^H NMR spectroscopy.

c *M*_n,theo_ were calculated using molecular weight of sodium methacrylate.

d *M*_n,abs_ and *Đ* were determined by size exclusion chromatography analysis with a multiangle light scattering detector (SEC-MALS) using aqueous NaCl/PBS (pH = 8) as the eluent.

e *M*_n,theo_/*M*_n,abs_.

**Figure 5:**
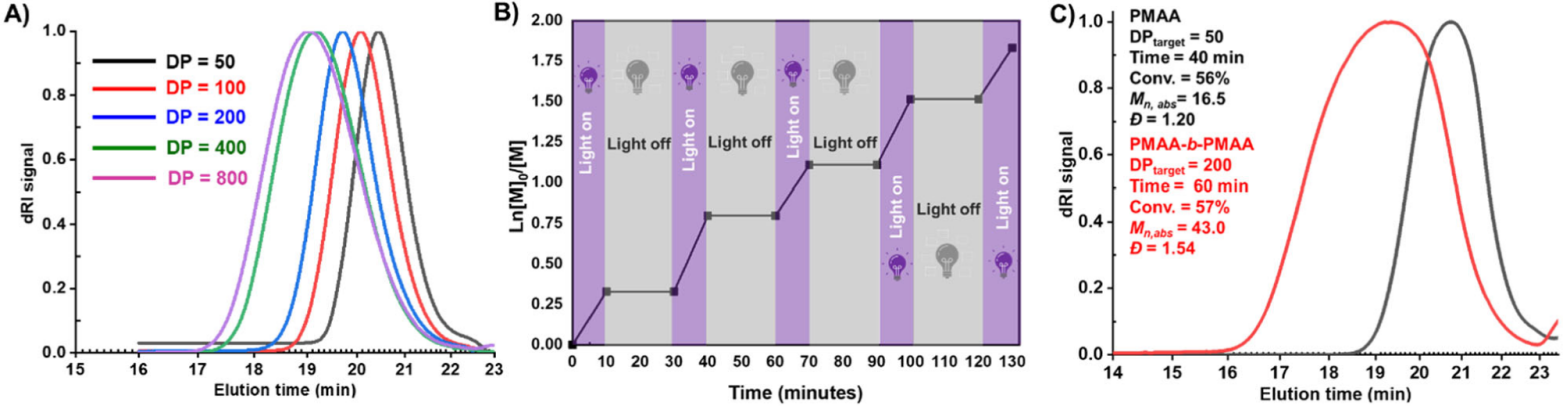
(A) PICAR-ATRP of MAA at different DP_target_ (i.e., 50, 100, 200, 400, 800); (B) temporal control over the polymerization of MAA, (C) *in situ* chain extension of MAA from a PMAA macroinitiator (DP_target_ = 50).

Temporal control of MAA polymerization was then demonstrated (Figure 5B). Temporal control is an important feature to prevent polymerization runaway, especially on larger industrial scales. Polymerization of MAA progressed during light irradiation without an induction time. After the removal of the light source, complete cessation of polymerization was observed during a 20-min period due to the rapid conversion of Cu^I^/L activator to Cu^II^/L deactivator by oxygen. Re-exposure to the light resumed polymerization. The on/off cycles were repeated several times, indicating efficient temporal control of the polymerization process.

The chain-end fidelity of PMAA synthesized by PICAR-ATRP was then investigated by chain extension experiments. Although the fast PICAR-ATRP reduced the extent of lactonization during polymerization, it could not completely prevent it in the resulting polymers, which contained CO_2_^–^ and halogen end groups. Therefore, chain extension experiments were carried out *in situ* without further purification. The polymerization for the first block was carried out using conditions reported earlier (entry 2, Table 3; 65 equiv of SP and DP_target_ = 100) and was stopped at 62% conversion. Then, the macroinitiators were used for chain extension without further purification by the addition of fresh MAA. Unfortunately, under these conditions , a bimodal SEC trace was observed, suggesting that some halogen chain ends were lost (Figure S7). As described earlier, the kinetics deviated from linear pseudo-first-order plots at high conversion, suggesting the occurrence of termination reactions and loss of chain-end fidelity. The very fast polymerization came at the expense of increased radical termination. By targeting a lower DP_target_ (50), using a lower SP concentration (16 equiv), and stopping the polymerization at lower conversion (56%), the chain-end functionality of PMAA was significantly improved. A shift of SEC traces to higher molecular weight was observed without significant tailing or bimodality (Figure 5C). Polymerization at lower SP concentration (16 equiv) for the synthesis of the first block at DP = 50 (Figure 5C) afforded PMAA with significantly lower dispersity compared to the results reported in Table 3. Although partially controlled behavior was observed by successful chain extensions, the molecular weight distributions of the second block were relatively broad (*Đ* = 1.54).

### Scale-Up and Low-Organic Content Synthesis of PMAA

Using the optimized reaction conditions with the CuCl_2_/TPMA complex and the CPAC initiator, ATRP of MAA was scaled up to 250 mL in the setup shown in Figure 6A. The polymerization was carried out in an open vessel with stirring at 250 rpm. The content of DMSO was reduced to 1% (2.5 mL), which was necessary to dissolve CuCl_2_ and TPMA. While DMSO is often used in photo-ATRP to scavenge singlet oxygen,^50,65^ other polar aprotic solvents (e.g., dimethylformamide (DMF)) could also solubilize the catalyst and be used instead of DMSO, resulting in a high conversion of MAA (Table S2). The scale-up polymerization was slower than that of smaller-volume polymerization, but it reached 50% conversion after 1.75 h of irradiation (370 nm, 100 mW/cm^2^). The semilogarithmic plot showed a linear increase over time, suggesting a constant concentration of radicals (Figure 6C). The temperature of the polymerization mixture increased by ca. 10 °C due to the exothermicity of the polymerization and the heat generated by light. 5.6 g of PMAA was purified by dialysis (cf. ^1^H NMR spectrum, Figure S8). The slight green color of the purified PMAA suggested the presence of some Cu coordinated to carboxylate groups from PMAA (Figure 6B), despite 72 h of purification of PMAA by dialysis in water. The addition of acid (HCl) during the dialysis purification reduced the binding of Cu to PMAA and formed white polymer products.

**Figure 6:**
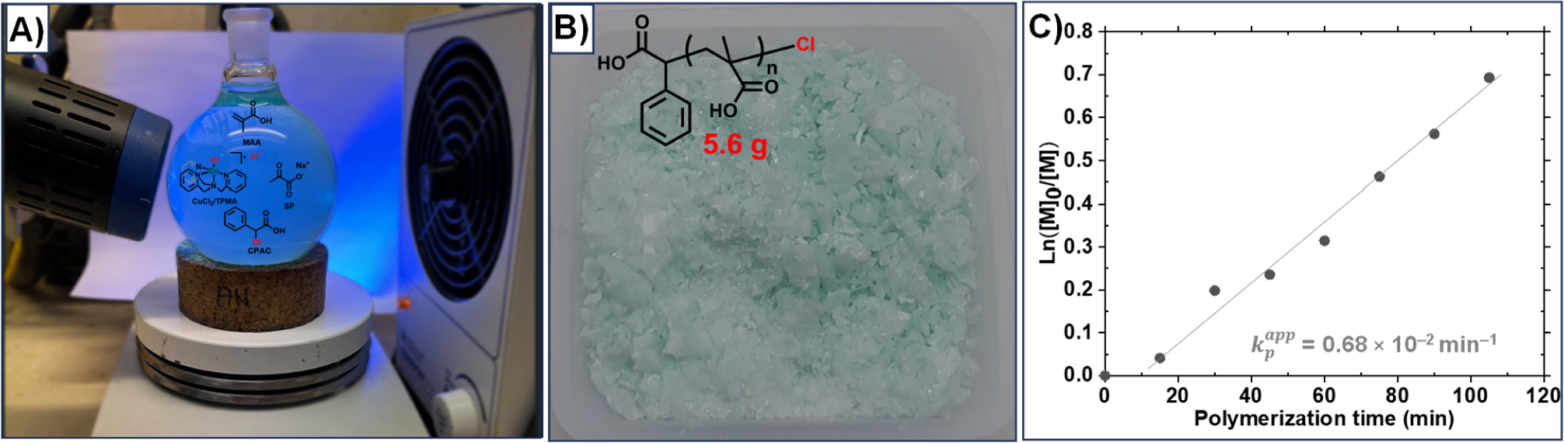
Scale-up polymerization of PMAA. (A) The setup for photo-ATRP of MAA with SP, CuCl_2_/TPMA, and CPAC initiator in a 250 mL round-bottom flask. (B) Digital image of purified PMAA after polymerization. (C) Kinetics of open-vessels and large-scale PICAR-ATRP of MAA. Conditions of polymerization: [MAA]_0_/[CPAC]_0_/[CuCl_2_]_0_/[TPMA]_0_/[SP]_0_ = 200/1/0.2/0.6/65 in H_2_O with DMSO (1% *v*/*v*) and PBS (10% *v/v*), irradiated under UV LEDs (370 nm, 100 mW/cm^2^).

### Synthesis of Branched P­(M)­AA

ATRP enables the preparation of polymers with diverse architectures and compositions, including star, (hyper)­branched, bottlebrush, and graft copolymers.^95–97^ Branched polymers, in contrast to their linear counterparts, show distinct properties, such as enhanced solubility, reduced intrinsic viscosities, and a high number of end-group functionalities.^98,99^ Recently, a new method for synthesizing branched polymers was reported using α-haloacrylates, also known as inibramers (IBM), which were copolymerized with various vinyl monomers (i.e., methacrylates, acrylates, acrylamides, and acrylonitrile) via ATRP.^100–103^ The higher bond dissociation energy of the C­(*sp* ^2^)–*X* bond in IBM compared to the C­(*sp* ^3^)–*X* bond in classical ATRP initiators (i.e., CPAC), maintained IBM inactive until it was incorporated into a polymer chain (Figure 7A), thereby allowing controlled formation of branching points.^104,105^ The ability of ATRP to generate branched PAA demonstrated an advantage of ATRP over RAFT polymerization for the synthesis of branched PMAA/PAA.

**Figure 7:**
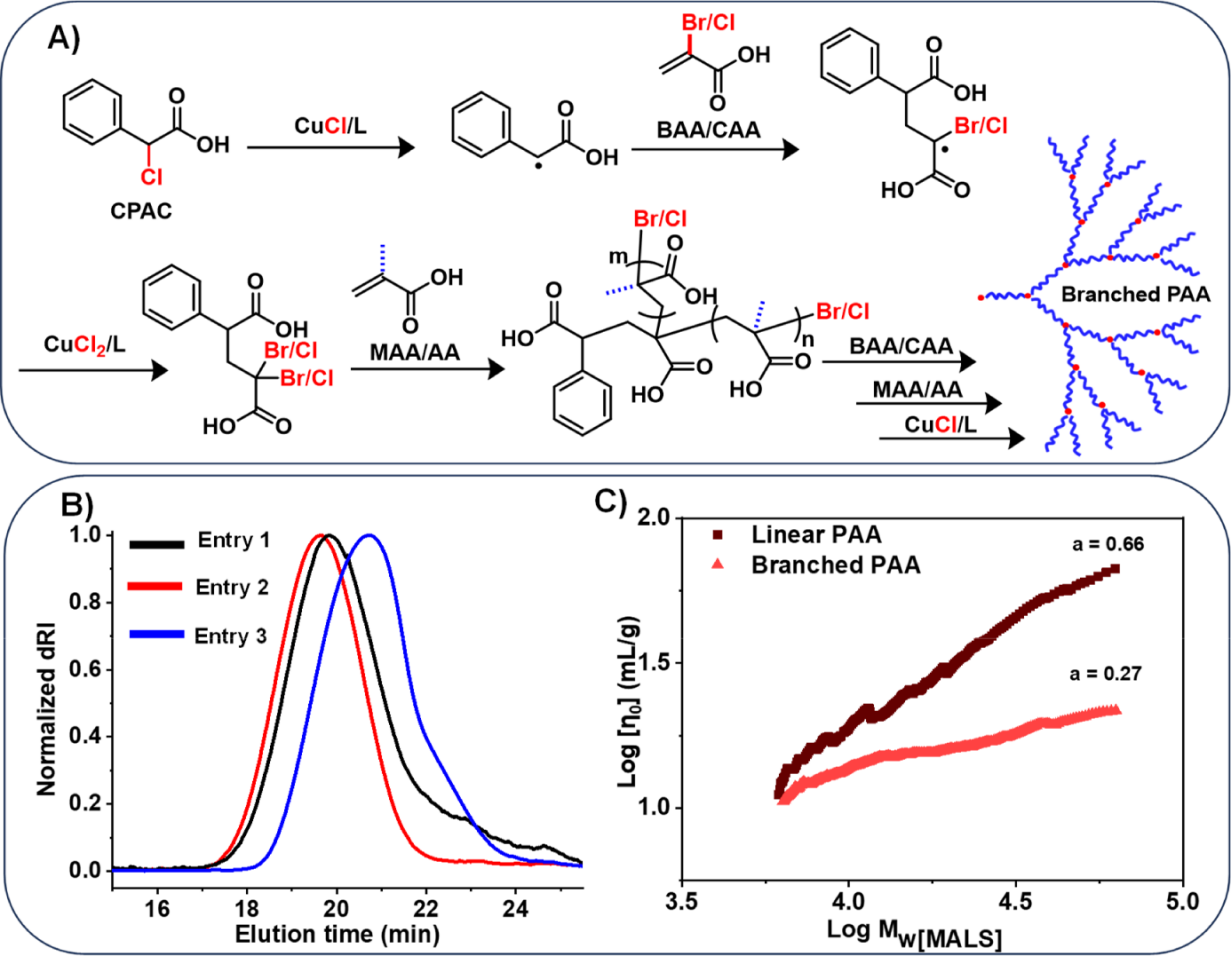
(A) Mechanism for the synthesis of branched P­(M)­AA in the presence of IBM. (B) SEC traces of branched PAA, presented in Table 4. (C) Comparison of Mark–Houwink–Sakurada plot of linear and branched PAA (entry 3, Table 4).

Herein, we applied this technique for the synthesis of well-defined branched PAA polymers. Commercially available 2-bromoacrylic acid (BAA) and 2-chloroacrylic acid (CAA) were used as the IBM and copolymerized with AA using the CPAC initiator (entries 1 and 2, Table 4) to form branched PAA with a molar ratio of [AA]_0_/[IBM]_0_/[CPAC] = 200/10/1. After 75 min of irradiation with UV light, IBM achieved higher conversions for both BAA and CAA (>99%) than AA (46% and 62%, respectively) (Figure S9), confirming the higher reactivity of IBM and the formation of gradient branched polymers.

**Table 4:** Branched PMAA and PAA Synthesized by Copolymerization of (M)­AA and BAA/CAA^a^

Entry	Monomer	IBM (mol %)	^b^Conv. (%) MAA/AA	^b^Conv. (%) IBM	^c^ *M*_n,theo__(×1000)_	^d^ *M*_n,abs__(×1000)_	^d^ *Đ*	^e^I* (%)
1	AA	BAA (5)	46	>99	10.4	7.8	1.47	133
2	AA	CAA (5)	62	>99	12.9	9.9	1.51	129
3^f^	AA	CAA (10)	56	>99	11.8	9.5	1.69	125
4	MAA	BAA (5)	65	90	15.6	7.6	1.47	204
5	MAA	CAA (5)	75	99	16.8	11.5	1.32	147

a Reactions conditions: [MAA/AA]_0_/[IBM]_0_/[CPAC]_0_/[CuCl_2_]_0_/[TPMA or Me_6_TREN]_0_/[SP]_0_ = 200/0−20/1/0.2/0.6/65 in H_2_O with DMSO (10% *v*/*v*) and PBS (10% *v/v*) irradiated under UV light (380 nm, 28.5 mW/cm^2^) at 1 mL scale without stirring at room temperature for 75 min. pH was adjusted to 1. [AA]_0_ or [MAA]_0_ = 1742 mM.

b Conversion was determined by ^1^H NMR.

c *M*_n,theo_ were calculated using molecular weight of sodium methacrylate/sodium acrylate.

d *M*_n,abs_ and *Đ* were determined by SEC analysis with a multiangle light scattering (MALS) using aqueous NaCl/PBS (pH = 8) as the eluent.

e *M*_n,theo_/*M*_n,abs_.

f CAA was fed into polymerization (feeding rate = 0.33 eq./min).

The resulting branched PAA (B-PAA) was then analyzed by size exclusion chromatography (SEC) with multiangle light scattering and viscosity detectors (MALS-VIS). Dispersity values were higher (*Đ* = 1.47 and 1.51) than those of the linear polymers prepared under the same conditions, and polymers with relatively higher *M*_n,theo_ than *M*_n,abs_ obtained, suggesting some IBM acts as initiator. While the SEC trace of B-PAA synthesized with BAA showed tailing in the lower molecular weight region (black trace), the SEC of B-PAA synthesized with CAA was monomodal and without tailing (red trace, Figure 7B). To achieve a higher degree of branching while ensuring statistical incorporation of IBM, CAA (10 mol %) was slowly fed into the polymerization reaction of AA (entry 3, Table 4). The resulting polymer revealed slightly better agreement between the *M*_n,abs_ and *M*_n,theo_, but still exhibited a broad molecular weight distribution (*Đ* = 1.69, blue trace, Figure 7B), which is a characteristic feature of hyperbranched polymers.^106,107^ The presence of branching was confirmed by examining the slopes (a) within the conformation plot (Mark–Houwink–Sakurada plot and log–log plot of [η] versus *M*_*w*_). The smaller slope of B-PAA (*a* = 0.27) versus L-PAA (*a* = 0.66) demonstrated the successful formation of branched polymers (Figure 7C).

The copolymerization of MAA with both BAA and CAA was also carried out. The SEC-MALS analysis of the resulting copolymers of MAA and BAA revealed significantly lower *M*_n, abs_ than *M*_n,theo_ (entries 4 and 5, Table 4), along with tailing in the low-molecular weight region (Figure S10). The tailing was attributed to β-carbon fragmentation of the PMAA backbone, as the midchain radicals generated upon halogen activation favored β-carbon fragmentation in polymethacrylates rather than branching.^91,108^ This suggests that the copolymerization of BAA/CAA with MAA was less successful in generating branched polymers than in the copolymerization with AA.

### Grafting PMAA from Fluoropolymer Using PICAR-ATRP in Water

Fluoropolymers, including poly­(vinylidene fluoride) (PVDF) and poly­(vinylidene fluoride-*co*-chlorotrifluoroethylene) (PVDF-*co*-CTFE), are extensively used for many applications due to their excellent chemical, physical, and thermal stability.^109–114^ Grafting hydrophilic polymers (i.e., AA, MAA, oligo­(ethylene oxide) methyl ether methacrylate, sulfopropyl methacrylate, styrene sulfonic acid, *N*-isopropylacrylamide, and 2-hydroxyethyl methacrylate) onto fluoropolymers is used to reduce their hydrophobicity and improve processability and performance.^115–118^ Organic solvents (typically *N*-methyl-2-pyrrolidone or DMSO) are used to dissolve monomers and polymers in spite of their very different solubility.^119–122^

As presented in Table 5, PICAR-ATRP of MAA was used for grafting MAA (DP_target_ = 10–40) from PVDF-*co*-CTFE (90/10 wt %) in water and DMSO (90%/10% *v/v*) in which PVDF has very limited solubility (Figure 8A). All polymerizations were carried out without any degassing, and PA was used as both a radical source and an oxygen scavenger. Activation of Cl in the PVDF-*co*-CTFE and subsequent grafting of hydrophilic PMAA by ATRP formed PVDF-*co*-CTFE-*g*-PMAA. As shown in Figure 8A, this gradually increased the dispersibility of graft copolymers in water.^123,124^ Upon irradiation of the polymerization mixture with UV light, the insoluble aggregates of PVDF-*co*-CTFE, dispersed in water with stirring, progressively changed to a stable emulsion-like system. A longer polymerization time (4 h) was required to reach high conversion (Figure 8B, entry 1, Table 5) compared to the homogeneous polymerization of PMAA, which was attributed to the slower activation of Cl in PVDF-*co*-CTFE macroinitiator versus small-molecule initiators (HOBiB or CPAC).

**Table 5:**
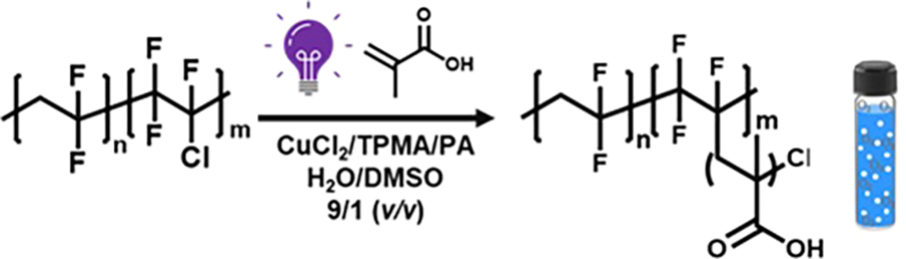
PICAR-ATRP of MAA from PVDF-*co*-CTFE in Water without Deoxygenation^a^

Entry	Monomer	Ligand	^b^DP	^c^Conv. (%)	^d^Weight of PMAA (mg)	^e^Weight of PVDF (mg)	Weight (%) PVDF/PMAA (%)
1	MAA	TPMA	10	82	200	33.9	14/86
2	MAA	TPMA	20	91	230	16.9	7/93
3	MAA	TPMA	40	68	170	8.4	5/95
4	AA	Me_6_TREN	20	<5	-	-	-

a Reactions conditions: [MAA]_0_/[PVDF-*co*-CTFE]_0_/[CuCl_2_]_0_/[TPMA]_0_/[PA]_0_ = 10–40/1/0.2/0.6/65 in H_2_O with DMSO (10% *v*/*v*) and PBS (10% *v/v*), irradiated under Evoluchem LEDs (380 nm, 28.5 mW/cm^2^) for 4 h in a closed-cap 1 dram vial (with stirring, 500 rpm). [MAA]_0_ = 580 mM. [PVDF-*co*-CTFE]_0_ = 1.45−5.8 mM.

b With respect to 10 Cl in PVDF-*co*-CTFE.

c Monomer conversion was determined by using ^1^H NMR spectroscopy.

d Calculated by multiplying conversion of MAA to the initial weight of MAA.

e Weight of PVDF-*co*-CTFE used in the feed.

**Figure 8:**
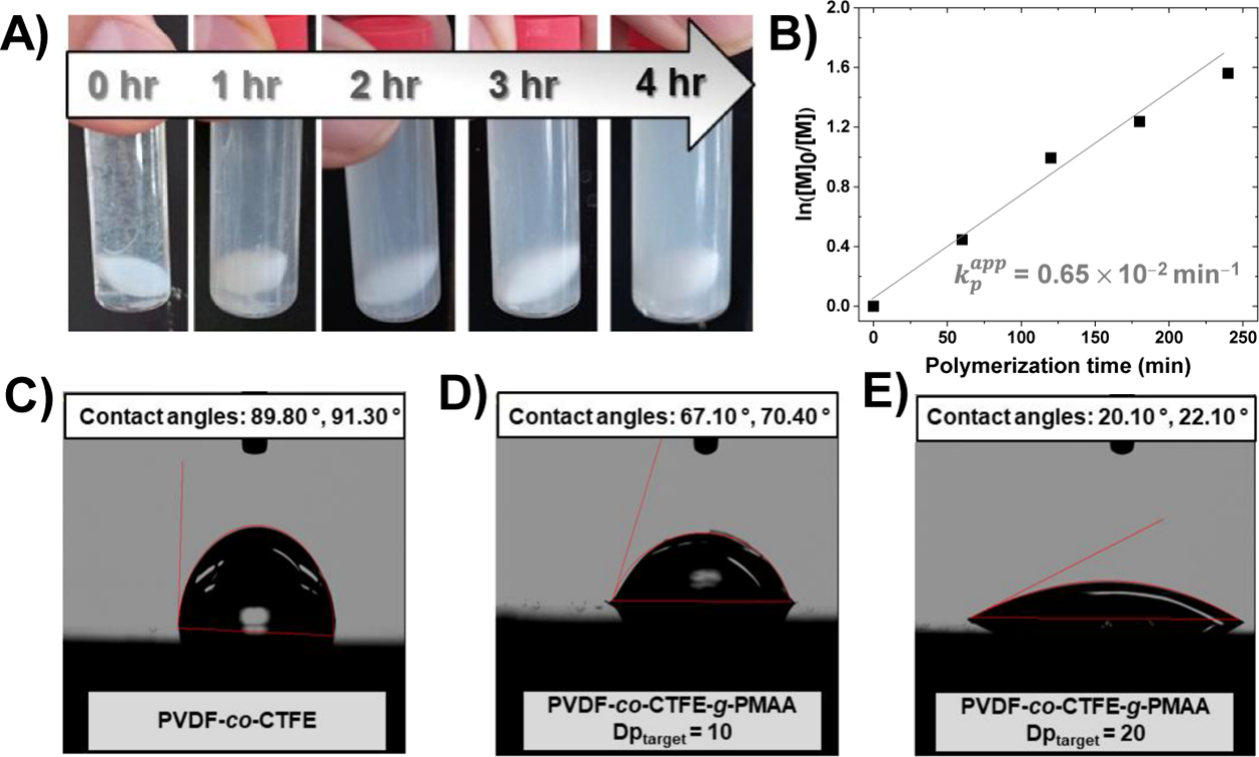
(A) Images of the progress of grafting polymerization of MAA from PVDF-*co*-CTFE (DP_target_ = 20) and (B) kinetics of PICAR-ATRP of MAA with PVDF-*co*-CTFE (DP_target_ = 20). Water contact angle measurements of (C) PVDF-*co*-CTFE, (D) PVDF-*co*-CTFE-*g*-PMAA (DP_target_ = 10), and (E) PVDF-*co*-CTFE-*g*-PMAA (DP_target_ = 20), cast on silicon wafer.

Purified polymers were then resuspended in water and exhibited very good dispersibility compared with the physical mixture of PMAA and PVDF-*co*-CTFE, which precipitated immediately in water (Figure S11), confirming the formation of graft copolymers. The improved dispersion of amphiphilic graft copolymers in water was further supported by dynamic light scattering (DLS, Figure S12). Successful grafting of PMAA was then proven by ^1^H NMR, with characteristic peaks of PMAA (0.8–1.2 and 12.3 ppm) and PVDF (2.9 ppm) (Figure S13). The FT-IR analysis aligned with the ^1^H NMR results, as the stretching bands of both PVDF and PMAA were present in the graft copolymers (Figure S14). The polymerization at lower or higher PMAA DPs was carried out and reached high conversions (entries 2 and 3, Table 5). PICAR-ATRP of AA with Me_6_TREN as a ligand did not afford any polymers after 5 h of UV irradiation (entry 4, Table 5). The significant change in the water contact angle of the graft copolymers (67.1 °, 70.4 ° for DP_target_ = 10; 20.1°, 22.1 ° for DP_target_ = 20), compared to PVDF-*co*-CTFE (89.8 °, 91.3 °), confirmed the formation of graft copolymers (Figure 8C–E).

The weight % (wt %) of PMAA grafted from PVDF by PICAR-ATRP was >85%. Therefore, this method is suitable for the high-density grafting of PMAA from PVDF (Table 5). A polymerization mixture with higher PVDF content (wt % > 14) or using higher molecular weight PVDF was not stable and precipitated.

## Conclusions

Cu-catalyzed photo-ATRP of MAA and AA was successfully carried out, yielding polymers with relatively narrow molecular weight distributions (*Đ* = 1.10–1.38 under optimized conditions) but limited initiation efficiency (I* % > 50% in most cases) at different pH values (1.2 and 3.2) in the presence of two common alkyl halide initiators (HOBiB and CPAC). Benefiting from the radical flux generated by the photodecomposition of sodium pyruvate or pyruvic acid, relatively fast polymerization at ambient temperature exhibited remarkable oxygen tolerance. This resulted in excellent temporal control and reduced intramolecular lactonization reactions, as evidenced by chain extension and the formation of well-defined polymers up to DP_target_ = 400. The extent of side reactions of MAA in photo-ATRP was negligible. The photo-ATRP of MAA with SP opened avenues for the development of new P­(M)­AA architectures, including branched and grafted copolymers from hydrophobic PVDF-*co*-CTFE in aqueous media.

## Supplementary Material



## Notes

Figure S1, ^1^H-NMR of MAA (bottom), sodium methacrylate (middle), and a mixture of MAA and sodium pyruvate (top); Figure S2, SEC traces of PMAA (left) and PAA (right) synthesized by PICAR-ATRP presented in Table 1 and Table 2; Figure S3, ATRP of MAA from PEG-Br macroinitiator (PEG-bromide, *M*_n_ = 2,166 g/mol); Figure S4, Analysis of photodecomposition (top) and ^1^H-NMR analysis (bottom) of SP in D_2_O upon UV light irradiation at different time (0–60 min); Figure S5, Possible mechanism for photoreductive cleavage of the mixture of CPAC-MAA; Figure S6, Decarboxylative electron transfer of MAA to CuCl_2_/TPMA: UV-VIS of CuCl_2_/TPMA solution under UV light irradiation with MAA (left) and without MAA (right); Figure S7, SEC traces of chain extension experiment of PMAA with MAA; Figure S8, ^1^H-NMR of PMAA from scale-up polymerization in D_2_O; Figure S9, The first-order kinetic plots for copolymerization of BAA with AA (A) and CAA with AA (B); Figure S10, SEC-MALS traces of polymerization of MAA and BAA; Figure S11, Digital images of the physical mixture of PVDF-*co*-CTFE and PMAA (left) and PVDF-*co*-CTFE-*g*-PMAA (right) in water/DMSO (8/2 v/v); Figure S12, Dynamic light scattering (DLS) of PMAA (black) and PVDF-*co*-CTFE-*g*-PMAA (red) in water; Table S1, Polymerization of MAA in the presence of different equivalents of SP; Table S2, Polymerization of MAA in the presence of different polar organic solvent­(PDF)
