# Supplementary material for: Open-Vessel and Scalable Synthesis of Linear and Branched Poly(meth)acrylic Acid via Light-Mediated Atom Transfer Radical Polymerization in Water
Source: Macromolecules. 2025 Jun 11;58(12):6190–202. doi: 10.1021/acs.macromol.5c00952 (PMC12199466; doi:10.1021/acs.macromol.5c00952)
Supplement: Supplementary file 1 [file ma5c00952_si_001.pdf]

Open Vessel and Scalable Synthesis of Linear and Branched Poly(meth)acrylic Acid via Light-mediated Atom Transfer Radical Polymerization in Water

*Arman Moini Jazani, Kriti Kapil, Hironobu Murata, Mozhdeh Madadi, Julian Sobieski, Piotr Mocny, Khidong Kim, Roberto R. Gil, and Krzysztof Matyjaszewski**

Department of Chemistry, Carnegie Mellon University, 4400 Fifth Avenue, Pittsburgh, PA 15213, United States.

Experimental Details

Materials

All chemicals were received from commercial vendors and used as received unless otherwise noted. Tris(2-pyridylmethyl) amine (TPMA, 97%) from Ambeed; water (HPLC grade), phosphate buffered saline (PBS, 10X with 1.37 M NaCl, 0.027 M KCl and 0.119 M phosphates), dimethyl sulfoxide (DMSO, ≥99.7%), 2-bromoacrylic acid (BAA, 95%), and 2-chloroacrylic acid (CAA, 96%, stabilized with 0.3% BHT) from Fisher Scientific; tris[2-(dimethylamino)ethyl]amine (Me_6TREN, 98%) from Alfa Aesar; sodium pyruvate (SP, >97%), pyruvic acid (PA, >97%) from Tokyo Chemical Industry; poly(vinylidene fluoride-*co*-chlorotrifluoroethylene) (PVDF-*co*-CTFE, 90/10) from PolyK Technologies; oligo(ethylene oxide) methyl ether methacrylate (average M_n = 500, $OEOMA_{500}$), copper (II) bromide ($CuBr_2$, 99.99%), copper (II) chloride ($CuCl_2$, 99%), 2-hydroxyethyl α-bromoisobutyrate (HOBiB, 95%), methacrylic acid (MAA, 99%), acrylic acid (AA, 99%), and α-chlorophenyl acetic acid (CPAC, 97%), from Sigma-Aldrich were purchased. MAA and AA were passed through neutral alumina before use. $OEOMA_{500}$ was passed through a column of basic alumina to remove the inhibitor prior to use.

Instrumentation

^{1}H-NMR spectra were recorded using a Bruker Avance III 500 MHz spectrometer. The D_2O was used as the NMR solvent.

Diffusion-filtered 1D ^{1}H-NMR (1D DOSY) experiments were performed on a Bruker NEO 500 MHz NMR instrument (operating at 500.00 MHz for ^{1}H) equipped with a multinuclear BBO

Prodigy cryoprobe, using the 1D DOSY pulse program ledbpgp2s1d from the Bruker pulse program library. The diffusion delay, little delta (p30), was set to 2,000 μs, and big delta (d20) to 100 ms. Gradient pulses were applied using the SMSQ10.100 gradient shape (integral factor of 0.9). The maximum Z-gradient strength of the probe is 65.7 Gauss/cm; however, considering the integral factor of 0.9 for the gradient shape SMSQ10.100, 100% gradient intensity corresponded to 59.13 Gauss/cm.

A series of 1D DOSY experiments with gradient pulse strengths gpz6 of 2% (1.18 Gauss/cm), 10% (5.91 Gauss/cm), 20% (11.83 Gauss/cm), 30% (17.74 Gauss/cm), and 40% (23.65 Gauss/cm) was performed on PMAA synthesized by CPAC initiator. The signal intensity corresponding to small molecules in the sample decreased as the gradient intensity increased, eventually disappearing entirely at 40% of gpz6, as shown in Figure 4.

Size exclusion chromatography (SEC) measurements of PMAA and PAA were performed using the Agilent SEC system (Agilent, 1260 Infinity II) coupled with ultra violet (UV, Agilent), multi angle light scattering (MALS), dynamic light scattering (DLS), Viscometer (VIS) and refractive index (RI) detectors (Wyatt Technology, USA). Measurements were performed using Suprema Lux 3000Å, 10 mm and guard columns with mixture of 100 mM NaCl and 10 mM sodium phosphate (pH = 8.0) as an eluent at room temperature and a 0.5 mL/min flow rate. Absolute molecular weight ($M_{n,abs}$) was determined by ASTRA 8.0.1 software (Wyatte Technology) using the dn/dc = 0.135 for both PMAA and PAA in the eluent. For the sample preparation, polymers were dialyzed in deionized water overnight, the clear solutions were filtered using a 0.22 mm nylon filter to remove any insoluble species and injected into the SEC.

The water contact angle on the surface was measured using VCA Optima system (AST products, Inc.) with a drop size 1.0 μL of deionized water. PVDF-*co*-CTFE and its graft copolymers were spin-coated on a silicon wafer. Three measurements at different spots were taken with each substrate, and the average of these values was determined.

Ultraviolet–visible (UV-Vis) spectra were recorded using an Agilent 8453 spectrophotometer.

UV light EvoluChem LEDs (λ_{max} = 380 nm, 28.5 mW/cm^2) and a Kessil LEDs (370 nm, 100 mW/cm^2) were used for polymerizations, and photoredox box with the in-built fan was purchased from Hepatochem.

General procedure for ATRP of MAA

For polymerization of MAA (entry 1, Table 2), MAA (0.15 g, 1.7 mmol), CPAC (2.9 mg, 17 μmol), TPMA (3.0 mg, 10.4 μmol), $CuCl_2$ (0.46 mg, 3.4 μmol), SP (124 mg, 1.1 mmol), DMSO (100 μL) and PBS (10X, 100 μL) were weighed into a volumetric flask (1 mL). Water was added to the flask to dissolve all the reagents and bring up the volume to 1 mL. After mixing the solution by vortexing for 2 minutes, the pH was reduced by the addition of HCl. The reaction mixture was irradiated by UV light in a photoredox box for 45 min. The polymerization was stopped by turning the light source off and diluting the polymerization mixture in the final characterization solvents (D_2O and H_2O). The samples were then analyzed by ^{1}H-NMR spectroscopy and SEC.

General procedure for polymerization for various target DPs

The target degrees of polymerization (DP_{target}) were varied by adding different amounts of CPAC to the polymerization mixture, while the concentrations of all the other components were maintained: $[MAA]_0$ = 1742 mM, $[CuCl_2]_0$ = 1.7 mM, $[TPMA]_0$ = 5.2 mM, $[SP]_0$ = 566 mM.

Polymerization mixtures (5 mL) were prepared by mixing MAA (0.75 g, 8.7 mmol), $CuCl_2$ (1.2 mg, 8.7 μmol), TPMA (7.6 mg, 26.0 μmol), SP (0.31 mg, 2.8 mmol), followed by the addition of DMSO (500 μL, 10% *v/v*), PBS (10X, 500 μL, 10% *v/v*) and water. After dividing the mixture into five different vials (1 mL), CPAC (2.2-35 μmol for DP_{target} = 50-800) were added using the stock solution. The mixtures were vortexed for 3 minutes, transferred to vials and irradiated with UV light.

General procedure for temporal control experiments

A mixture of MAA (0.75 g, 8.7 mmol), $CuCl_2$ (1.2 mg, 8.7 μmol), TPMA (7.6 mg, 26.0 μmol), SP (0.31 mg, 2.8 mmol), CPAC (7.4 mg, 43.6 μmol) was prepared and followed by the addition of DMSO (500 μL, 10% *v/v*), PBS (500 μL, 10% *v/v*) and water. After vortexing the solution, the vial was placed into photo-reactor. The reactor was exposed to periodic irradiation phases that each lasted for around 10 min and stopped for 20 min for each interval. The aliquots (75 μL) were withdrawn after each irradiation or dark phase and analyzed by ^{1}H NMR.

General procedure for chain extension experiments

The *in-situ* chain extension reported in Figure 5C was carried out by mixing MAA (0.75 g, 8.7 mmol), $CuCl_2$ (1.2 mg, 8.7 µmol), TPMA (7.6 mg, 26.0 µmol), SP (0.30 g, 2.8 mmol), CPAC (29.7 mg, 0.17 mmol), PBS (10X, 500 µl) in a volumetric flask (5 mL). After adjusting the DMSO volume (500 µL, 10% *v/v*), the volumetric flask was topped up with water. The mixture was vortexed, transferred to an open-cap glass vial (1 mL), and irradiated with light (40 min). Polymerization was stopped by turning the light off, and aliquot of polymerization (500 µl) was mixed with a fresh MAA (0.30 g, 3.5 mmol) in another glass vial (1 mL). The mixture was vortexed and irradiated with UV light for another 1 hour.

General procedure for polymerization of $OEOMA_{500}$ via photoactivation of CPAC and MAA mixture

A mixture of $OEOMA_{500}$ (0.75 g, 1.5 mmol), MAA (38.7 mg, 0.45 mmol), CPAC (2.5 mg, 15 µmol), DMSO (500 µL) was prepared in a volumetric flask (5 mL). Water was added to the flask to dissolve all the reagents and make up the volume to 5 mL. The mixture was transferred to a vial (1 mL) and irradiated with UV light for 2 hours.

Procedure for scale-up ATRP of MAA

For scale up polymerization of MAA in a 250 mL round bottom flask, MAA (37.5 g, 0.43 mol) $CuCl_2$ (58.0 mg, 0.43 mmol) and TPMA (0.38 g, 1.31 mmol) were dissolved in DMSO (2.5 mL), followed by CPAC (0.37 g, 2.18 mmol), SP (15.6 g, 0.14 mol) and PBS (10X, 25 mL). Water was added to the flask to dissolve all the reagents and fill up the volume to 250 mL. After mixing the solution by stirring for 10 minutes, the reaction mixture was stirred (250 RPM) and irradiated by Kessil UV light (370 nm, 100 mW/cm^2) for 105 minutes with a fan to cool down the temperature. The polymerization was stopped by turning the light source off and diluting the polymerization mixture in the final characterization solvents (D_2O and H_2O). The samples were then analyzed by ^{1}H-NMR spectroscopy and SEC. The polymers were purified by dialysis in water for 72 hours.

General procedure for synthesis of branched PAA (B-PAA)

For synthesis of B-PAA (entry 3, Table 4), AA (0.62 g, 8.60 mmol), $CuCl_2$ (1.16 mg, 8.6 μmol), Me_6TREN (5.9 mg, 25.8 μmol), CPAC (7.3 g, 43 μmol), SP (0.31 g, 2.8 mmol), DMSO (500 μL) and PBS (10X, 500 μL) were weighed into a volumetric flask (5 mL). Water was added to the flask to dissolve all the reagents and make up the volume to 5 mL. The reaction mixture was vortexed for 2 minutes and was transferred to a vial (1 mL). CAA (18.5 mg, 0.17 mmol) was dissolved in DMF and fed into the reaction mixture (1 mL) at the rate 0.33 equivalent/min, while it was irradiated with UV light in a photoredox box for 75 minutes. The polymerization was stopped by turning the light source off and diluting the polymerization mixture in the final characterization solvents (D_2O and H_2O). The samples were then analyzed by ^1H-NMR spectroscopy and SEC.

General procedure for grafting MAA from PVDF-*co*-CTFE

For the synthesis of PVDF-*co*-CTFE-*g*-PMAA (entry 1, Table 5), PVDF-*co*-CTFE (34 mg, 29 μmol) was dissolved in DMSO (500 μL, 10% *v/v*) in a glass vial (5 mL). MAA (0.25 mg, 2.9 mmol), $CuCl_2$ (0.78 mg, 5.8 μmol), TPMA (5.0 mg, 17.4 μmol), PA (0.16 mg, 1.9 mmol) and PBS (10X, 500 μl) was then added to vial and was topped up with water. The solution was vortexed for 3 minutes. After closing the cap, the vial was placed into photo-reactor and was stirred at 500 RPM and then irradiated for 4 hours. The aliquots (75 μL) were withdrawn dissolved in DMSO-d_6 and analyzed by ^1H-NMR. Polymers were purified by dialysis in water overnight.

Comparing solubility of graft and physical mixture PVDF-*co*-CTFE with PVDF-*co*-CTFE-*g*-PMAA

PVDF-*co*-CTFE (12 mg) was dissolved in DMSO (2 mL) followed by adding water (8 mL) and PMAA (20 mg). For graft copolymers, PVDF-*co*-CTFE-*g*-PMAA (32 mg) was added to DMSO (2 mL) and followed by adding water (8 mL) and vortexed for 5 minutes.

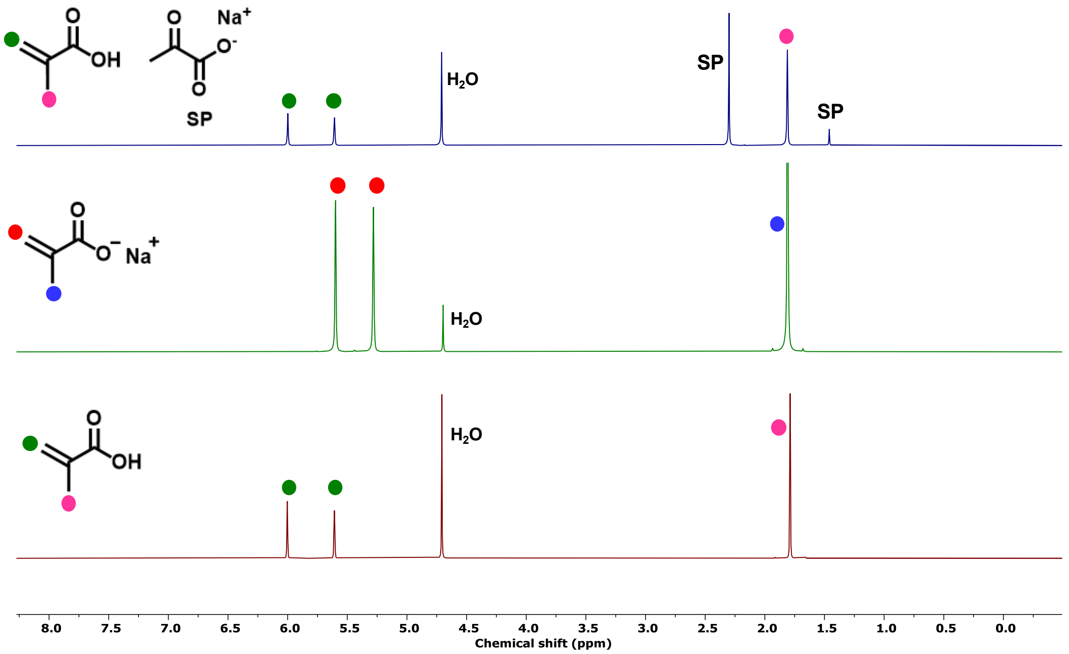

Figure S1. ^{1}H-NMR of MAA (bottom), sodium methacrylate (middle), and a mixture of MAA and sodium pyruvate (top). Sodium pyruvate is in equilibrium with 2,2-dihydroxypropanoate.

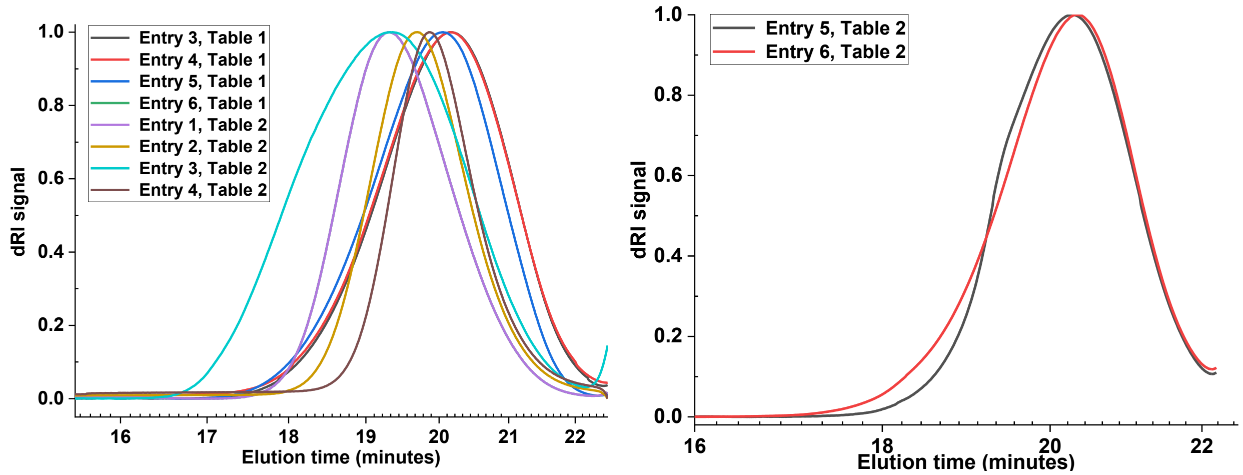

Figure S2. SEC traces of PMAA (left) and PAA (right) synthesized by PICAR ATRP presented in Table 1 and Table 2.

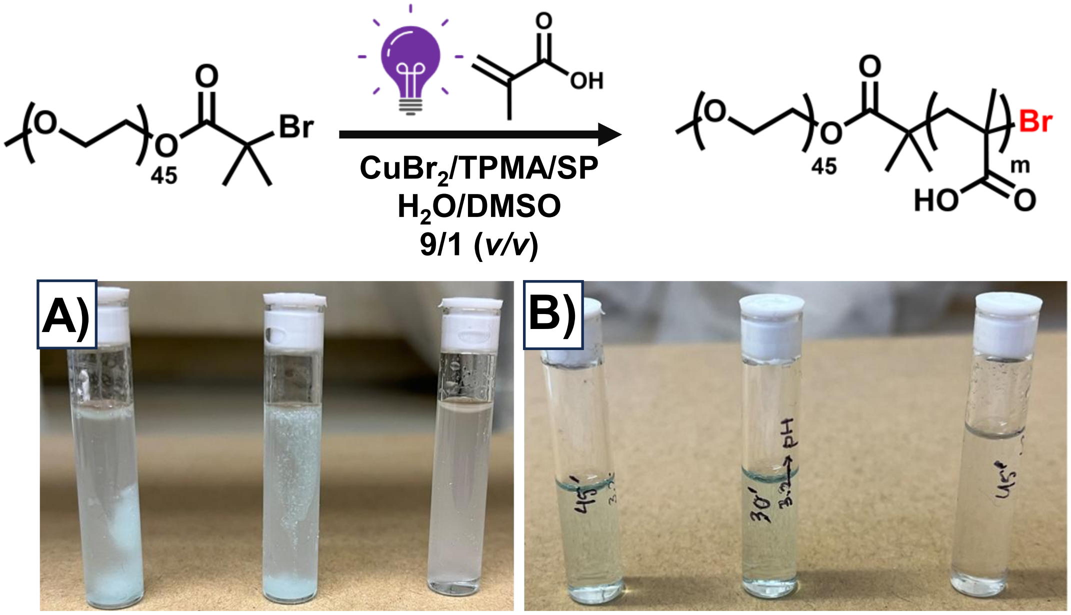

Figure S3. ATRP of MAA from PEG-Br macroinitiator (PEG-bromide, M_n = 2,166 g/mol). (A) Precipitates formed during polymerization after light irradiation; (B) redissolved polymers in water after the addition of sodium bicarbonate.

Table S1. Polymerization of MAA in the presence of different equivalence of SP.[a)]

Entry	SP (eqv)	Time	[b]Conv. (%)
1	8	3	70
2	8	6	98
3	16	1	40
4	16	2	58
5	16	3	76
6	64	0.75	72

[a)] Reactions conditions: $[MAA]_0/[CPAC]_0/[CuCl_2]_0/[TPMA]_0/[SP]_0$ = 200/1/0.2/0.6/8-64 in H_2O with DMSO (10% *v/v*) and PBS (10% *v/v*), irradiated with Kessil UVs (370 nm, 28.5 mW/cm^2) in an open cap vial at 1 mL scale (without stirring). $[MAA]_0$ = 1742 mM. [b)] Monomer conversion was determined by using ^{1}H-NMR spectroscopy.

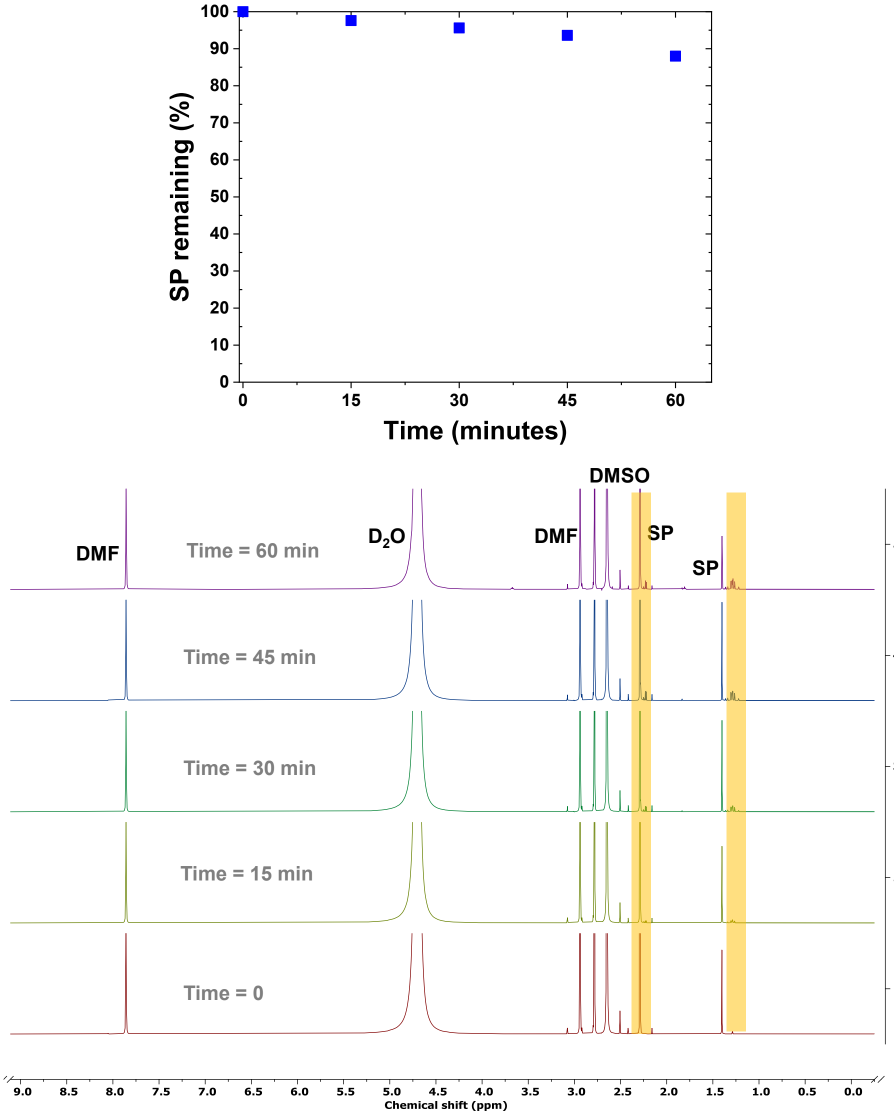

Figure S4. Analysis of photodecomposition (top) and ^{1}H-NMR analysis (bottom) of SP in D_2O upon UV light irradiation at different time (0-60 min). Conditions of experiment: $[SP]_0 = 566$ mM in D_2O and 10% *v/v* of DMSO and PBS. 50 μL of DMF was used as standard.

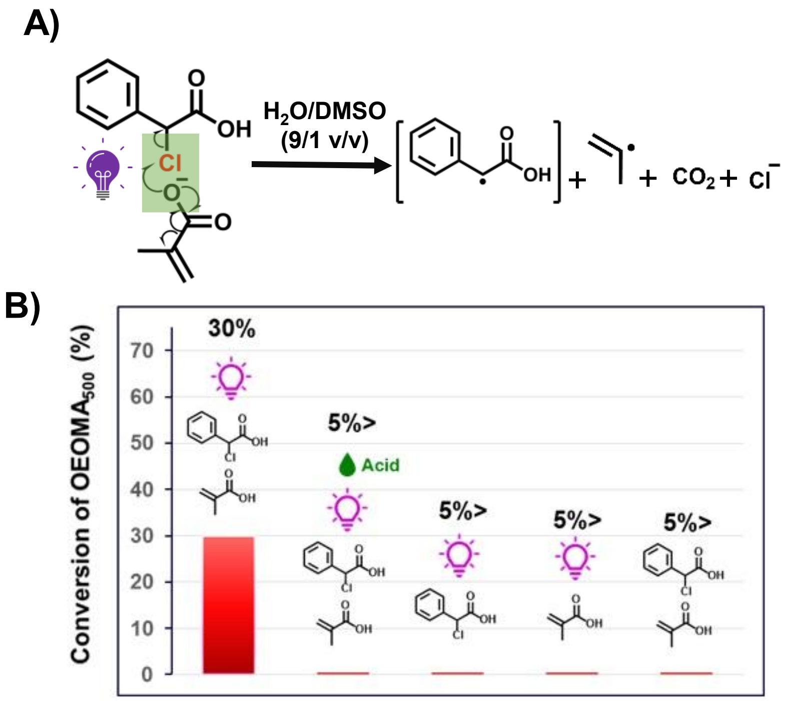

Figure S5. (A) possible mechanism for photo reductive cleavage of the mixture of CPAC-MAA; (B) conversion of $OEOMA_{500}$ measured by ^{1}H-NMR for demonstrating radicals' generation. Reactions conditions: $[OEOMA_{500}]_0/[MAA]_0/[CPAC]_0$ = 100/30/1 in H_2O with DMSO (10% v/v), irradiated with UV light for 2 hours. $[OEOMA_{500}]_0$ = 300 mM.

To quantify alkyl halide activation with carboxylic acids upon light irradiation, an experiment was devised using oligo(ethylene oxide) methacrylate ($OEOMA_{500}$), and conversion of $OEOMA_{500}$ was monitored for the MAA ratio which reduced to 30 mol % in the feed. A mixture of MAA, $OEOMA_{500}$, and CPAC resulted in 30% conversion of $OEOMA_{500}$ after UV irradiation and generated viscous polymer solutions (Figure S5). The conversion of the carboxylate anions (COO^-) to carboxylic acids (COOH) by the addition of HCl (pH = 1) inhibited this reaction. Other control experiments confirmed the need of the CPAC, MAA, and UV light for this reaction.

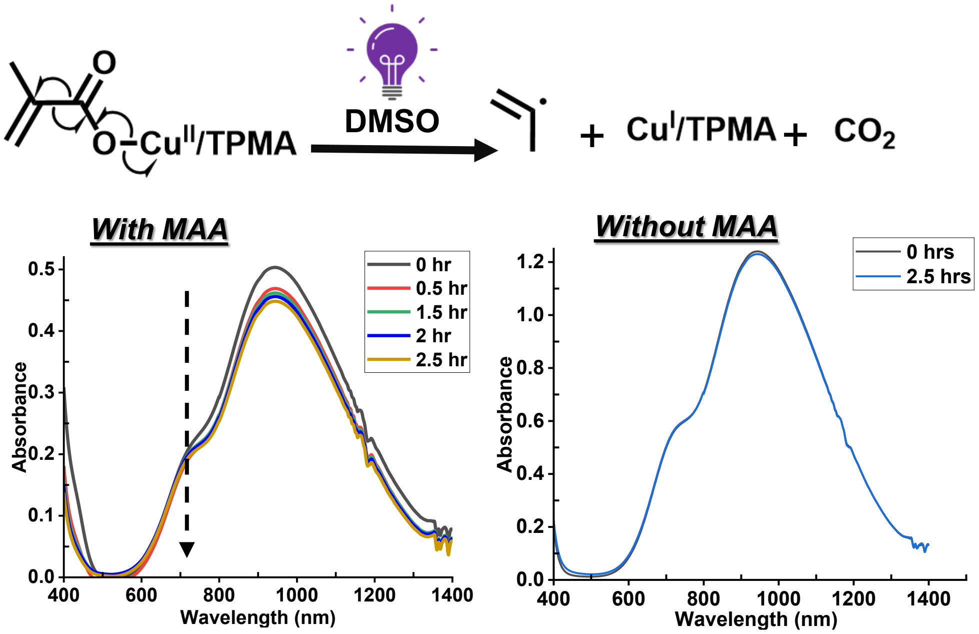

Figure S6. Decarboxylative electron transfer of MAA to $CuCl_2$/TPMA: UV-VIS of $CuCl_2$/TPMA solution under UV light irradiation with MAA (left) and without MAA (right).

To investigate the coordination of MAA with $CuCl_2$/TPMA complex and subsequent photodecarboxylation of MAA with UV light, the UV-VIS spectra of a mixture of $CuCl_2$/TPMA (1 equiv.) and MAA (20 equiv.) in DMSO were recorded after UV light irradiation and compared with the control solution without MAA. As seen in Figure S6, the spectra of the MAA-$CuCl_2$/TPMA mixture show a decrease in absorption within 2.5 hours irradiation, suggesting MAA promoted light-induced photoreduction of $CuCl_2$, whereas no change in the spectra was observed without MAA. The reduction of Cu^{II} suggests the electron transfer from MAA, which subsequently leads to the homolytic cleavage of the carbon–carbon bond in MAA, the release of CO_2, and formation of radicals. Such unstable radical species can be deactivated by $CuCl_2$, form new polymer chains, or react with available oxygen in the media. Nevertheless, the extent of reaction after 1.5 hours of light irradiation was only 8 %, according to the UV-VIS spectra. Furthermore, there was no change in SEC traces of PMAA after 2 hours of UV irradiation in the presence of $CuCl_2$/TPMA complexes, suggesting the negligible effect on ATRP.

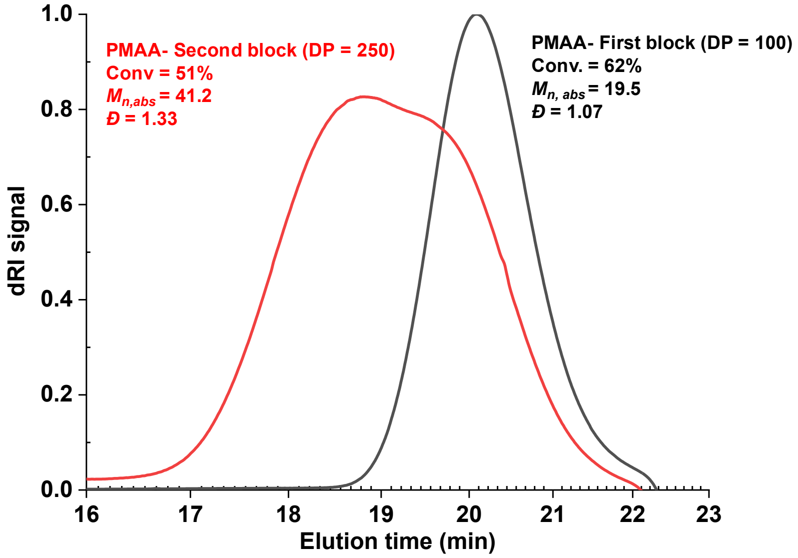

Figure S7. SEC traces of chain extension experiment of PMAA with MAA. Conditions of polymerization for first block: $[MAA]_0/[CPAC]_0/[CuCl_2]_0/[TPMA]_0/[SP]_0$ = 100/1/0.2/0.6/65 in H_2O with DMSO (10% *v/v*) and PBS (10% *v/v*), irradiated under UV LEDs.

Table S2. Polymerization of MAA with PICAR ATRP in the presence of different polar organic solvent.[a)]

Entry	Solvent	CuX_2	[b]Conv. (%)
1	DMSO	$CuBr_2$	72
2	DMF	$CuBr_2$	45
3	MeCN	$CuBr_2$	0

[a)] Reactions conditions: $[MAA]_0/[HOBiB]_0/[CuX_2]_0/[TPMA]_0/[SP]_0$ = 200/1/0.2/0.6/65 in H_2O with DMSO, DMF, or MeCN (10% *v/v*) and PBS (10% *v/v*), irradiated with Kessil UV (370 nm, 28.5 mW/cm^2) in an open cap vial at 1 mL scale (without stirring) for 45 mins. $[MAA]_0$ = 1742 mM. [b)] Monomer conversion was determined by using ^{1}H-NMR spectroscopy.

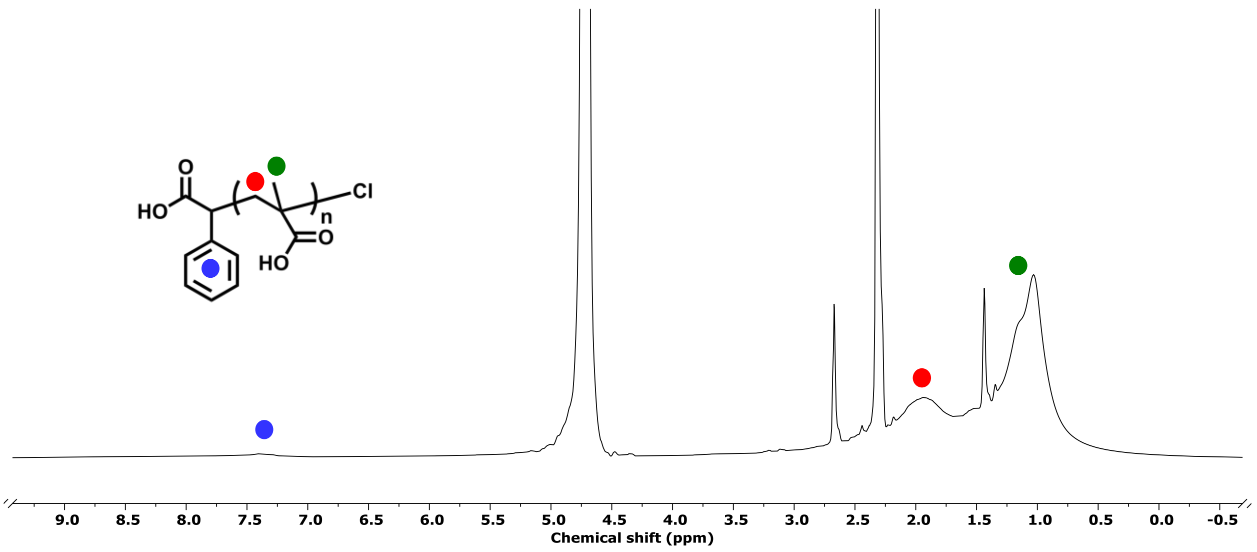

Figure S8. ^{1}H-NMR of PMAA from scale-up polymerization in D_2O.

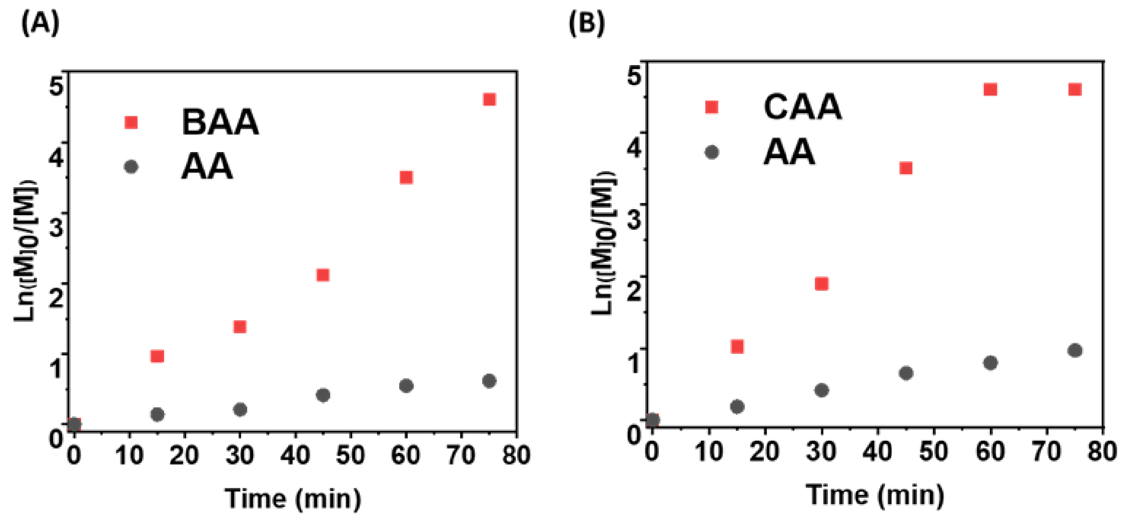

Figure S9. The first-order kinetic plots for copolymerization of BAA with AA (A) and CAA with AA (B).

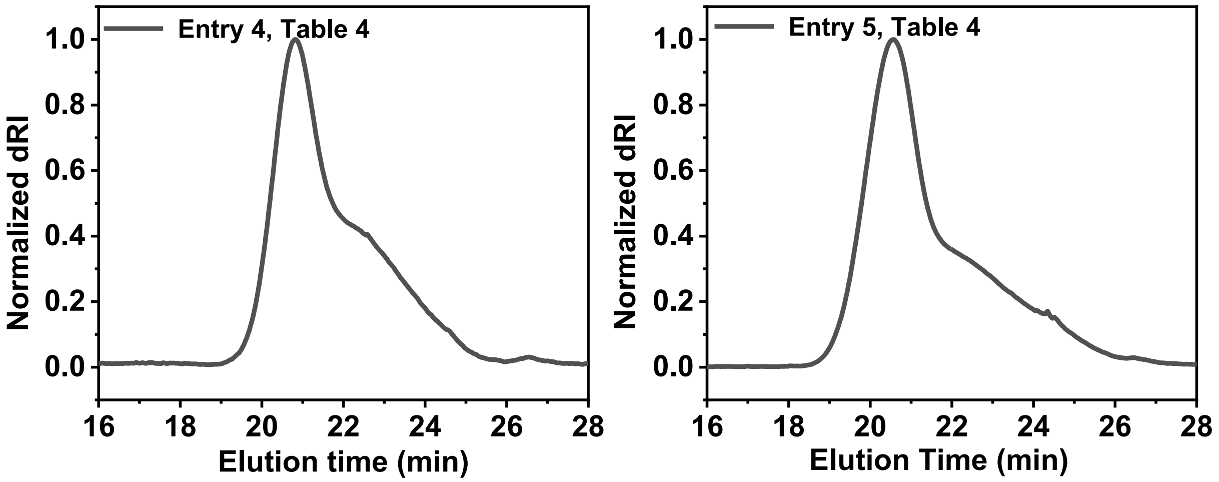

Figure S10. SEC-MALS traces of polymerization of MAA and BAA.

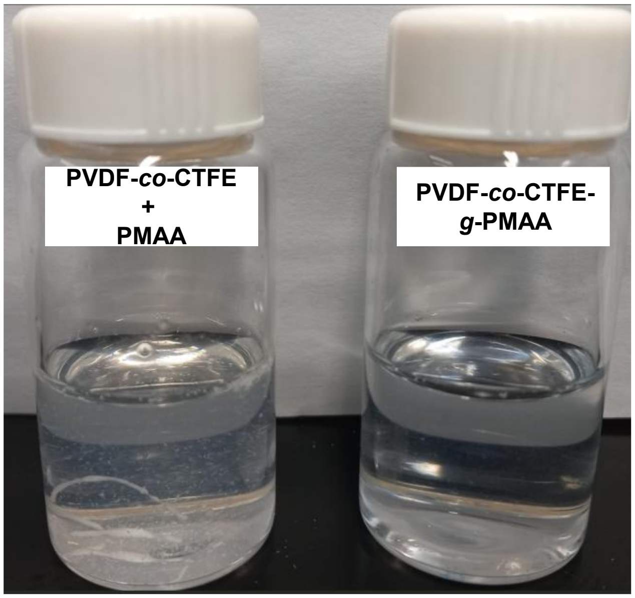

Figure S11. Digital images of the physical mixture of PVDF-*co*-CTFE (1.2 mg/mL) and PMAA (left) and PVDF-*co*-CTFE-*g*-PMAA (3.2 mg/mL, right) in water/DMSO (8/2 *v/v*).

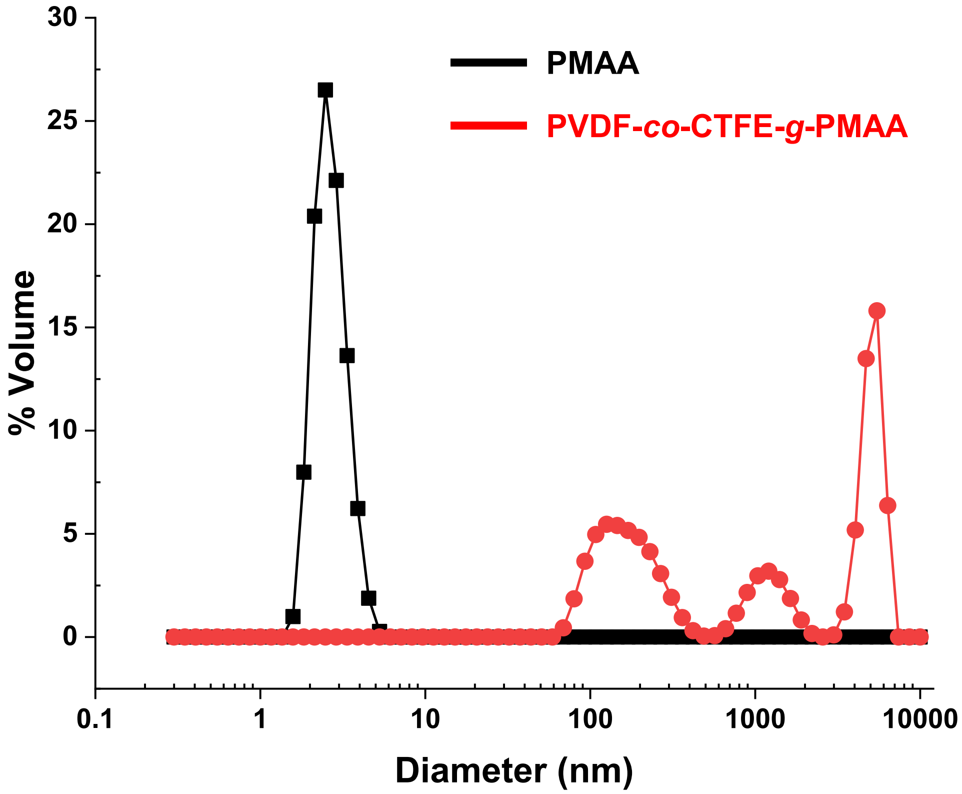

Figure S12. Dynamic light scattering (DLS) of PMAA (black) and PVDF-*co*-CTFE-*g*-PMAA (red) in water. The formation of aggregates indicates the formation of graft copolymers. PVDF-co-CTFE precipitated in water before measurement could be made.

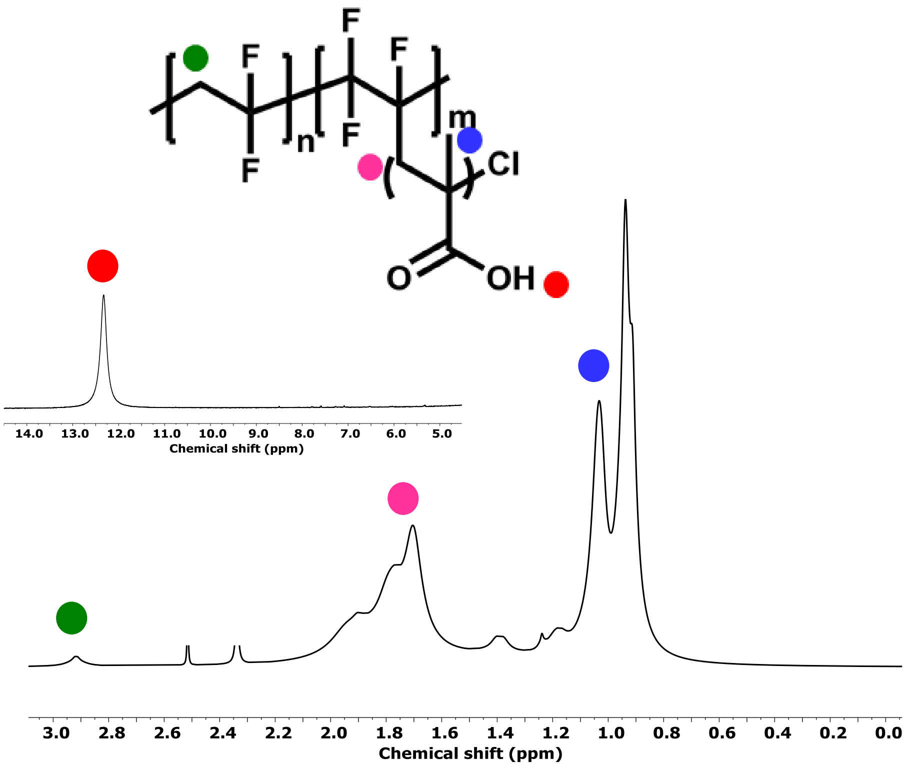

Figure S13. ^{1}H-NMR of purified PVDF-*co*-CTFE-*g*-PMAA in DMSO-d_6 (entry 1, Table 5).

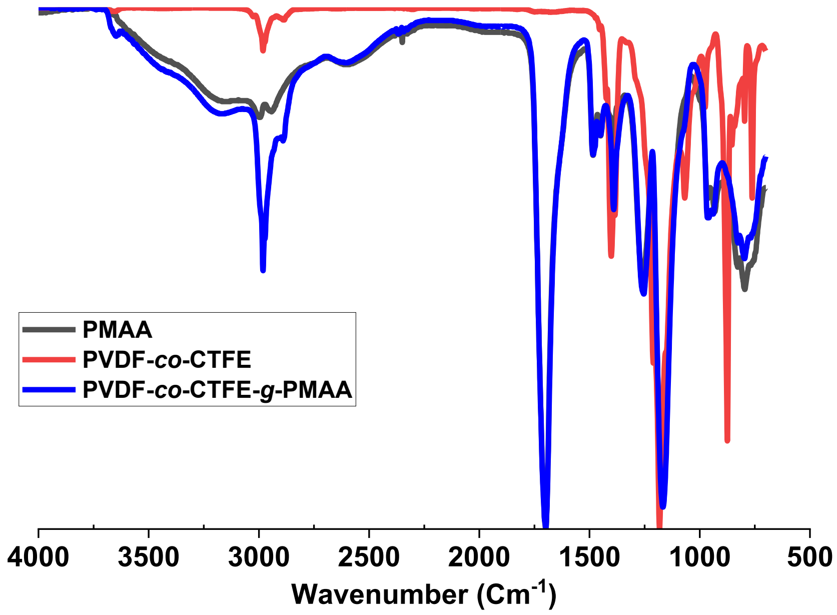

Figure S14. FT-IR of PMAA (black), PVDF-*co*-CTFE (red) and PVDF-*co*-CTFE-*g*-PMAA (blue) graft copolymers (entry 1, Table 5).
